# Twenty‐first century adaptive teaching and individualized learning operationalized as specific blends of student‐centered instructional events: A systematic review and meta‐analysis

**DOI:** 10.1002/cl2.1017

**Published:** 2019-07-19

**Authors:** Robert M. Bernard, Eugene Borokhovski, Richard F. Schmid, David I. Waddington, David I. Pickup

**Affiliations:** ^1^ Centre for the Study of Learning and Performance Concordia University Montreal Canada; ^2^ Centre for the Study of Learning and Performance Montreal Canada; ^3^ Department of Education (Educational Technology), Centre for the Study of Learning and Performance Concordia University Montreal Canada; ^4^ Department of Education (Educational Studies), Centre for the Study of Learning and Performance Concordia University Montreal Canada; ^5^ Centre for the Study of Learning and Performance Montreal Canada

## 1 PLAIN LANGUAGE SUMMARY

**Adaptive teaching and individualization for K‐12 students improve academic achievement**

### 1.1 The review in brief

Teaching methods that individualize and adapt instructional conditions to K‐12 learners’ needs, abilities, and interests help improve learning achievement. The most important variables are the teacher's role in the classroom as a guide and mentor and the adaptability of learning activities and materials.

**What is the aim of this review?**

This Campbell systematic review assesses the overall impact on student achievement of processes and methods that are more student‐centered versus less student‐centered. It also considers the strength of student‐centered practices in four teaching domains.

**Flexibility:** Degree to which students can contribute to course design, selecting study materials, and stating learning objectives.

**Pacing of instruction:** Students can decide how fast to progress through course content and whether this progression is linear or iterative.

**Teacher's role:** Ranging from authority figure and sole source of information, to teacher as equal partner in the learning process.

**Adaptability:** Degrees of manipulating learning environments, materials, and activities to make them more student‐centered.

### 1.2 What is this review about?

Teaching in K‐12 classrooms involves many decisions about the appropriateness of methods and materials that both provide content and encourage learning.

This review assesses the overall impact on student achievement of processes and methods that are more student‐centered versus less student‐centered (and thus more teacher‐centered, i.e., more under the direct control of a teacher). It also considers in which instructional dimensions the application of more of these student‐centered practices is most appropriate, and the strength of student‐centered practices in each of four teaching domains.

### 1.3 What is this review about?

#### 1.3.1 What studies are included?

This review presents evidence from 299 studies (covering 43,175 students in a formal school setting) yielding 365 estimates of the impact of teaching practices. The studies spanned the period 2000–2017 and were mostly carried out in the United States, Europe, and Australia.

*What is the overall average effect of more versus less student‐centered instruction on achievement outcomes? Which demographic variables moderate the overall results?*

More student‐centered instructional conditions have a moderate positive effect on student achievement compared to less student‐centered.

*Which dimensions of instruction are most important in promoting better achievement through the application of more versus less student‐centered instruction? Do these dimensions interact?*

The teacher's role has a significantly positive impact on student achievement; more student‐centered instruction produces better achievement. Pacing of instruction/learning—where learners have more choice over setting the pace and content navigation of learning activities—has a significant effect in the opposite direction; i.e., a significantly negative relationship. There is no relationship between adaptability and flexibility and student achievement.

There are interactive effects. The teacher's role combined with adaptability produces stronger effects, whereas flexibility (greater involvement of students in course design and selection of learning materials and objectives) has the opposite effect; it reduces the effectiveness of teacher's role on learning outcomes.

Special education students perform significantly better in achievement compared to the general population.

Three other factors—grade level; Science, technology, engineering, and mathematics (STEM) versus non‐STEM subjects; individual subjects—do not have any effect on the impact of the intervention.

### 1.4 What do the findings of this review mean?

This review confirms previous research on the effectiveness of student‐centered and active learning. It goes further in suggesting the teacher's role promotes effective student‐centered learning, and excessive student control over pacing appears to inhibit it.

An important element of these findings relates to the significant combination of teacher's role and adaptability, in that it suggests the domain in which the teacher's role should focus.

Since adaptability relates to increasing the involvement of students in more student‐centered activities, the evidence suggests that instruction that involves activity‐based learning, either individually or in groups, increases learning beyond the overall effect found for more student‐centered versus less student‐centered activities.

Various student‐centered approaches, such as cooperative learning and peer‐tutoring, have been found to accomplish this goal.

### 1.5 How up‐to‐date is this review?

This meta‐analysis contains studies that date from 2000–2017.

## 2 EXECUTIVE SUMMARY/ABSTRACT

### 2.1 Background

The question of how to best deliver instruction to k‐12 students has dominated the educational conversation, both in terms of theory and practice, since before 1960. Two predominant models have clashed: (a) Traditional teacher‐directed instruction (referred to here as teacher‐centered *Teacher‐Centered* instruction), where there is little methodological adaptation for individual differences in ability, skills, interests, etc. among students; and (b) so‐called student‐centered instruction (referred to here as *Student‐Centered* instruction), deriving much of its theoretical justification and methodological intricacies from constructivist thought embodied in the works of Jean Piaget, Lev Vygotsky, Jerome Burner, and many others. While radical constructivism has never become dominant in k‐12 schooling (except in a relatively small number of demonstration schools), there has been considerable interest in embedding some of the principles of constructivism into k‐12 schooling. This is often referred to as *individualized* or *adaptive* instruction, meaning an operational concern for individual students, their abilities, interests, etc., which is nearly the opposite of *Teacher‐Centered* instruction. A great deal of research has demonstrated that approaches to individualism, such as mastery learning, collaborative and cooperative learning, problem‐based learning, peer tutoring, and computer‐based instruction, are effective in promoting achievement and attitudinal gains, as contrasted with *Teacher‐Centered* instruction, where mastery of content or subject matter is of the greatest concern, and the teacher is the “delivery mechanism.” More recently, this has been extended to include video‐based lectures often delivered through the internet, as proposed by proponents of blended learning and its variant the flipped classroom (e.g., Baepler, Walker, and Driessen (2014). Research has also demonstrated that *Teacher‐Centered* instruction is particularly useful in developing basic skills in areas such as reading, spelling, and math (Stockard, Wood, Coughlin, & Khoury, 2018).

More recent theory and practice concerning *Teacher‐Centered* (more conventional) and *Student‐Centered* (more adaptive and individualized) instruction suggest that neither perspective is entirely sufficient and that some combination of *Teacher‐Centered* and *Student‐Centered* instruction is possibly more productive. This notion of combined teaching methods (i.e., *Teacher‐Centered* plus *Student‐Centered*) is one of the defining characteristics of the flipped classroom (Baepler et al., 2014). Certainly, students need to acquire skills and knowledge, but they also need to develop their own personal preferences, creativity, problem‐solving abilities, and evaluative and self‐evaluative perspectives. The current meta‐analysis aims to determine if the advantage endowed by *Student‐Centered* instruction also affects content achievement (i.e., content achievement is the outcome measure in this meta‐analysis).

The current meta‐analysis was designed to explore teaching and learning in k‐12 classrooms and the achievement benefit that derives from more *Student‐Centered* versus less *Student‐Centered* classrooms. Several perspectives informed the basis for the research approach described here, but none more so than the words of Gersten et al. (2008) while exploring through meta‐analysis the question of *Teacher‐Centered* versus *Student‐Centered* instructional practices in elementary mathematics instruction. In the final report of their study, the group stated: “The Task Group found no examples of studies in which learners were teaching themselves or each other without any teacher guidance; nor did the Task Group find studies in which teachers conveyed … content directly to learners without any attention to their understanding or response. The fact that these terms, in practice, are neither clearly nor uniformly defined, nor are they true opposites, complicates the challenge of providing a review and synthesis of the literature …” (p. 12). The current meta‐analysis intends to investigate variations of *more versus less Student‐Centered* instruction and the four domains of the instructional process in which they are more or less profitable.

### 2.2 Objectives (research questions)

There are three primary objectives that this meta‐analysis intends to address (research questions that this study explores):

- Overall, does *more Student‐Centered* instructional practices lead to a significant advantage in the acquisition of content (subject matter) knowledge (i.e., measured learning achievement)?
- Do any of the four primary (substantive) moderator variables (entered into multiple meta‐regression), *Teacher's Role*, *Pacing*, *Adaptability*, and *Flexibility*, predict an increase or decrease in achievement across degrees of *Student‐Centered* use (From *less Student‐Centered* to *more Student‐Centered*)?
- Is there a difference in categorical levels of *less Student‐Centered* to *more Student‐Centered* for each of the dimensions of instructional practice listed above, tested in mixed moderator variable analysis?
- Do any of the secondary (demographic) moderator variables interact with each other (i.e., combine) to produce more versus less *Student‐Centered* instructional practices?

### 2.3 Search methods

Following the guidelines of the Campbell Collaboration (Kugley et al., 2017), in order to retrieve a broad base of studies to review, we started by having an experienced Information Specialist search across an array of bibliographic databases, both in the subject area and in related disciplines. The following databases were searched for relevant publications: ABI/Inform Global (ProQuest), Academic Search Complete (EBSCO), ERIC (EBSCO), PsycINFO (EBSCO), CBCA Education (ProQuest), Education Source (EBSCO), Web of Knowledge, Engineering Village, Francis, ProQuest Dissertations & Theses Global, ProQuest Education Database, Linguistics and Language Behavior Abstracts (ProQuest).

The search strategy was tailored to the features of each database, making use of database‐specific controlled vocabulary and search filters, but based on the same core key terms. Searches were limited to the year 2000–2017 and targeted a k‐12 population.

Database searching was supplemented by using the Google search engine to locate additional articles, but principally grey literature (research reports, conference papers, theses, and research published outside conventional journals).

### 2.4 Selection criteria

The overall set of inclusion/exclusion criteria (i.e., selection) for the meta‐analysis contained the following requirements:

- Be publicly available and encompass studies from 2000 to the present;
- Feature at least two groups of different instructional strategies/practices that can be compared according to the research question as *Student‐Centered* and *Teacher‐Centered* instruction;
- Include course content and outcome measures that are compatible with the groups that form these comparisons;
- Contain sufficient descriptions of major instructional events in both instructional conditions;
- Satisfy the requirements of either experimental or high‐quality quasi‐experimental design;
- Be conducted in formal k‐12 educational settings eventually leading to a certificate, diploma, degree, or promotion to a higher grade level;
- Contain legitimate measures of academic achievement (i.e., teacher/researcher‐made, standardized); and
- Contain sufficient statistical information for effect size extraction.

### 2.5 Data collection and analysis

#### 2.5.1 Effect size extraction and calculation

One of the selection criteria was “Contain sufficient statistical information for effect size extraction,” so that an effect size could be calculated for each independent comparison. This information could take several forms (in all cases sample size data were required):

- Means and standard deviations for each treatment and control group;
- Exact *t* value, *F* value, with an indication of the ± direction of the effect;
- Exact *p* value (e.g., *p* = .011), with an indication of the ± direction of the effect;
- Effect sizes converted from correlations or log odds ratios;
- Estimates of the mean difference (e.g., adjusted means, regression β weight, gain score means when *r* is unknown)
- Estimates of the pooled standard deviation (e.g., gain score standard deviation, one‐way *ANOVA* with three or more groups, *ANCOVA*);
- Estimates based on a probability of a significant *t* test using α (e.g., *p* < .05); and
- Approximations based on dichotomous data (e.g., percentages of students who succeeded or failed the course requirements).

Effect sizes were initially calculated as Cohen's *d* (Cohen, 1988) and then converted to Hedges'g (i.e., correction for small samples; Hedges & Olkin, 1985). Standard errors (*SE*_*d*_) were calculated for *d* and then converted to standard errors of *SE*_*g*_ applying the correction formula for *g*. Hedges’ *g*, *SE*_*g*_, and sample sizes (i.e., treatment and control) were entered into *Comprehensive Meta‐Analysis* 3.3.07 (Borenstein, Hedges, Higgins, & Rothstein, 2014) where statistical analyses were performed.

The effect sizes were coded for precision and these data were analyzed in moderator variable analysis.

#### 2.5.2 Statistical analyses

Analyses were conducted using the following statistical tests:

- Overall weighted random effects analysis with the statistics of $\bar{g}$, *SE*_*g*_, *V*_g_, upper and lower limits of the 95th confidence interval, *z*_*g*_, and *p* value;
- Homogeneity is estimated using *Q*‐Total, *df*, and *p* value. *I* ^2^ (i.e., percentage of error variation) and tau^2^ (i.e., average heterogeneity) is also calculated and reported.
- Meta‐regression (single and multiple) is used to determine the relationship between covariates and effect sizes; and
- Mixed‐model (i.e., random and fixed) moderator variable analysis is used to compare levels (categories) of each coded moderator variable. *Q‐*Between, *df*, and *p* value are used to make decisions about the significance of each categorical variable.

### 2.6 Results

The results are presented here in relationship to the four research questions previously described.

- **Question 1:** Overall, does *more Student‐Centered* instructional practices lead to a significant advantage in the acquisition of content (subject matter) achievement (i.e., measured learning).
- **Result:** Answering the basic question, *more Student‐Centered* instructional conditions (i.e., the treatment described above) outperform *less Student‐Centered* to a moderate extent. The average effect, $\bar{g}$  = 0.44, *k* = 365, *z* = 4.56, *p* < .00, *SE* = 0.03, *Q* = 3,095.89, *I* ^2^ = 88.22, *tau* ^2^ = 0.27, between the mean of the more *Student‐Centered* treatment and the less *Student‐Centered* control, suggesting that teachers who promote and enact active classroom processes (more *Student‐Centered* instruction), can expect to see better student achievement than in classrooms where teachers employ less *Student‐Centered* instruction. Also, a linear trend was found in meta‐regression when Hedges’ $\bar{g}$was regressed on *degree of Student‐Centered instruction* (*β* = 0.04, *SE* = 0.02, *z* = 2.41, *p* = .032). The distribution remains significantly heterogeneous.
- **Question 2:** Do any of the four moderator variables (entered into multiple meta‐regression), *Teacher's Role*, *Pacing*, *Adaptability*, and *Flexibility*, predict an increase or decrease in achievement across degrees of *Student‐Centered* use (From *less Student‐Centered* to *more Student‐Centered*)?
- **Result:** In meta‐regression, *Teacher's role* produces a significant linear trend (*β* = 0.06, *SE* = 0.04, *z* = 4.42, *p* < .001) and *Pacing* (*β* = −0.14, *SE* = 0.04, *z* = 3.18, *p *= .002). *Adaptability,* and *Flexibility* are not significant (*p* > .05). However, the trend for *Teacher's role* and *Pacing* is opposite (note the opposite signs on *β*). *Teacher's role* is significantly positive (i.e., more *Student‐Centered* instruction produced higher achievement), while *Pacing* produces the reverse (i.e., a significantly negative trend). For *Pacing,* more *Student‐Centered* methods produce lower achievement.
- **Question 3:** Do any of the moderator variables interact with each other (i.e., combine) to produce more versus less *Student‐Centered* instructional practices?
- **Result:** Yes, *Teacher's Role* compared to two dimensions added to the *Teacher's Role* produce significantly different results (*Q*‐Between = 7.76, *df*  = 3, *p* = .02: *Teacher's Role* and *Teacher's Role* plus *Adaptability* significantly outperformed *Teacher's Role* plus *Flexibility*.
- **Question 4:** Is there a difference in categorical levels of *less Student‐Centered* to *more Student‐Centered* for each of the dimensions of instructional practice listed above, tested in mixed moderator variable analysis?
- **Result:** Only one of five moderator variables produced a significant differentiation among levels. Among four moderator variables (i.e., grade level; STEM versus Non‐STEM subjects; individual subjects; and ability profile) only *ability profile* significantly differentiated among levels. *Special education* students demonstrated significantly higher achievement compared to the *General population* of students.

### 2.7 Authors’ conclusions

This meta‐analysis provides strong evidence that *Student‐Centered* instruction leads to improvements in learning with k‐12 students. Not only is the overall random effects average effect size of medium strength ($\bar{g}$ = 0.44), but there is also a demonstrated (subtle but significant) linear relationship between more *Student‐Centered* classroom instruction and effect size (*p* = .03). Taken together, these results support the efficacy of allowing students to engage in active learning or other forms of *Student‐Centered* enterprise as part of a comprehensive educational experience.

## 3 BACKGROUND

### 3.1 Adaptive teaching and individualization for k‐12 students improve academic achievement: A meta‐analysis of classroom studies

The question of how to provide the best‐quality instructional conditions for students of all grade levels has been scrutinized extensively since the early 1960s, principally from two major perspectives: Teacher‐centeredness (*Teacher‐Centered*) and student‐centeredness (*Student‐Centered*). *Student‐Centered* education initially arose from the writings of early progressive educators like John Dewey, and was carried on subsequently, in various forms, by Jean Piaget, Lev Vigotsky, Jerome Bruner, and Carl Rogers, to name only a few. The ideas were radical when first introduced, but the notion of *Student‐Centered* education resonated in educational circles, where lecturing and rote memorization was still the standard for quality education and led to vast amounts of theorizing and research to show that students could succeed in learning of all sorts without a strongly transmissive approach on the part of the teacher. Today, the terms *individualized instruction* and *adaptive teaching* have become a popular expression for current practice and are used nearly synonymously with *Student‐Centered* learning.

However, since their inception, *Student‐Centered* practices have inspired resistance, both from the public and from educational theorists. Thus, after *Student‐Centered* practices were widely introduced, a dichotomy arose in the literature, with one side promoting the continuation of *Teacher‐Centered* learning and on the other side the adopting *Student‐Centered* learning practices. This was argued as a dichotomy for many years. However, the arguments have abated somewhat now with the general recognition that there is value in both approaches. Generally speaking, educators no longer aspire to a pure implementation of either approach, but now discuss questions of which method, when, and for what purpose is best.

#### 3.1.1 Individualized learning and adaptive student‐centered education (*Student‐Centered*)

Conceptual understanding of individualized learning and adaptive teaching varies broadly, encompassing a multitude of instructional strategies, approaches, and activities. It stretches from accounts of specific systems of instruction such as mastery learning (Bloom, 1968) and scaffolded adaptive feedback in computer‐based instruction (e.g., Azevedo & Bernard, 1995) to more general conceptions of active learning and individualization that involve approaches such as cooperative learning (e.g., Johnson & Johnson, 2002; Johnson, Johnson, & Maruyama, 1983), collaborative learning (e.g., Bernard, Rojo de Rubalcava, & St‐Pierre, 2000), problem‐based learning (e.g., Zhang et al., 2015), and project‐based learning (e.g., Bernard & Lundgren‐Cayrol, 2001). It also includes educational concepts, largely derived from elements of constructivism, such as discovery learning, inquiry‐based learning, activity‐based learning, experiential learning, and other forms of *Student‐Centered* education (Tobias & Duffy, 2009).

Notions of unguided *Student‐Centered* learners have not been free from detractors. Dewey criticized this approach in *Experience and Education* (Dewey, 1938), and, more recently, Kirschner, Sweller, and Clark (2006) published an influential piece that argued that the practice of turning kids loose to learn defies many of the tenets of the psychological principles of working memory and that guided instruction is both more efficient and ultimately more profitable to long‐term learning outcomes. A flurry of responses and rejoinders ensued with no clear resolution, but the educational community was left with the strong impression that a teacher's role in *Student‐Centered* learning was better as a *guide on the side* rather than a *silent witness* (King, 1993).

The learning sciences have further contributed to the distinction between social constructivism and individual constructivism providing a theoretical grounding for teacher versus learner‐based strategies (Kolodner, 2004). Current and developing applications, informed by pedagogical principles espoused by case‐based learning (e.g., Kolodner et al., 2003).

##### Research on more individualized and adaptive education

The earliest large‐scale research project, aimed at exploring the efficacy of so‐called progressive education, was conducted between 1933 and 1941 by the Progressive Education Association (funded by the General Education Board and other foundations). Twenty‐nine model schools were selected for curricular experimentation with the security that over 200 colleges and universities would accept their students upon recommendation by their principals. Changes in these schools included more individualized instruction and more access to alternative and cross‐disciplinary programs, which emphasized greater access to arts and extracurricular programs.

Results indicated that students graduating from the 200 schools scored on par in basic courses (e.g., mathematics and science) with students from traditionally oriented schools and that there was more activity in artistic, political, and social engagement in students from the alternative experimental schools. The long‐term impact of these experiments is generally described as influence on its participants and subsequent reformers rather than dramatic change. The intervening conservatism brought about by World War II and the ensuing Cold War are often cited as deterrents to widespread change in the overall educational system in the United States (Aiken, 1942).

Examples of further attempts to make teaching and learning more individualized and adaptive can be found in both the early and current research literature. They include, but are not limited to, mastery learning (e.g., Bloom, 1968), Personalized System of Instruction (PSI; e.g., Keller, 1968), assorted forms of peer instruction (e.g., Mazur, 1997), various practices of reciprocal reading/writing activities (e.g., Huang & Yang, 2015), collaborative and cooperative learning, problem and project‐based learning and, more recently, Intelligent Tutoring Systems (ITS; e.g., Huang & Shiu, 2012). Several of these approaches are summarized in the following paragraphs and a number of the most common group‐based *Student‐Centered* approaches are depicted in a Venn Diagram (Figure 2) that shows their inter‐relationship and approximate overlap (Bishop & Verleger, 2013, p. 6).

The benefits and limitations of so‐called systems of instruction (i.e., mastery learning, PSI, and ISI) are summarized separately in both qualitative and quantitative reviews. In the late 1970s and early 1980s, several relevant meta‐analyses were published on mastery learning and its variant PSI. First, Lysakowski and Walberg (1982), Guskey and Gates (1986), Guskey and Pigott (1988), Slavin (1987), and Kulik, Kulik, and Bangert‐Drowns (1990) each performed successive meta‐analyses (Slavin's was the best evidence synthesis) on the efficacy of mastery learning. The studies produced equivocal and highly debatable findings. Kulik, Kulik, and Cohen (1979) reviewed 75 individual comparative studies of Keller's Personalized System of Instruction (PSI is a spin‐off of mastery learning) college teaching method. In comparison to conventional instruction, the PSI approach was demonstrated to have a positive effect on student achievement and course perception (mean effect size of nearly 0.70*sd* for both).

Bangert and Kulik (1982) looked at the effectiveness of the Individualized Systems of Instruction (ISI, a spin‐off of PSI) in secondary school students. They broadened the list of outcomes to account not only for student achievement (e.g., final exams), but also critical thinking, attitudes toward subject matter, and student self‐concept. For all outcome types, the findings were inconclusive. For example, for the achievement data, only 8 out of 49 studies demonstrated statistically significant results in favor of ISI (four studies favored more conventional teaching methods and the rest were inconclusive). Finally, Kulik (1984) attempted a wider research synthesis (encompassing over 500 individual studies) of the effectiveness of programmed instruction and ISI, paying special attention to the moderator variables of study dates and grade levels. The most promising findings indicated that more recent studies showed higher effects than the earlier ones and that college‐level students benefited significantly from using ISI compared with elementary and secondary school students. In summary, as stated earlier, these meta‐analyses produced inconclusive results. Moreover, they are rather outdated and practically none of the above‐mentioned instructional methods exists now in their original forms (e.g., Eyre, 2007 was able to identify fewer than 50 studies of PSI for the period between 1990 and 2006 in the PsycInfo database).

Much of the preceding discussion has been about systems of individualized instruction, designed and intended as self‐contained approaches to individualizing student learning. Because of their rule‐based nature, they may be thought to be individualized, but insufficiently adaptive (systems often are not very adaptive).

Several meta‐analyses addressed the topic of individualized *and* adaptive instruction (i.e., instructional approaches that can be applied as local circumstances dictate), though in very specific narrowly focused forms. Aiello and Wolfle (1980) summarized research on individualized instruction in science education compared with traditional lectures and found that individualized instruction was more effective. Horak's (1981) meta‐analysis of self‐paced modular instruction of elementary and secondary school math (1981) produced a wide variety of both positive and negative effect sizes. A highly cited meta‐analysis of active learning in science, engineering, and mathematics subject matters (Freeman et al., 2014) found a moderate effect size ($\bar{d}$ = 0.47) based on 158 studies. The authors also state that “The results raise questions about the continued use of traditional lecturing as a control in research studies, and support active learning as the preferred, empirically validated teaching practice in regular classrooms” (p. 8410). This sentiment appears to add support to the comparative approach that is employed in the current meta‐analysis.

Kraft, Blazar, and Hogan (2018) examined the effects of teacher coaching (i.e., tutoring) on student achievement and found minor effect on achievement ($\bar{d}$ = 0.08). Though these instructional approaches are not “adaptive,” per se, at least peer tutoring opens the educational process to much greater involvement of students, and thus accounts more for their individual inputs in learning. The effect size tended to be relatively small in middle school students, but higher at elementary and high school levels.

There have been numerous reviews and meta‐analyses of various forms of computer‐assisted instruction (CBI). Ma, Adesope, Nesbit, and Liu (2014) meta‐analyzed studies of ITS in a variety of subject matters, from reading and math to law and medical education. More specific reviews have been conducted on the effectiveness of feedback and scaffolding in CBI and ITS. The list of moderator variables included the type of both experimental and comparison treatments, as well as outcome type, student academic level, study discipline, etc. The highest achievement effects of using ITS were found in comparison with non‐ITS computer‐based instruction ($\bar{d}$ = 0.57) and teacher‐centered, large‐group instruction ($\bar{d}$ = 0.42), whereas in comparison with human tutoring it was even negative ($\bar{d}$ = −0.11), though not statistically significant. ITS‐based practices were similarly effective when used either alone or in combination with various forms of teacher‐led instruction in many subject domains. In particular, certain specific aspects of instruction like feedback and scaffolding in CBI and ITS systems have come under scrutiny. Azevedo & Bernard (1995) examined studies testing the effectiveness of computer‐provided feedback against no feedback, and Belland, Walker, Olsen, and Leary (2015) synthesized studies investigating feedback in computer‐based scaffolding. In both cases, the average effect size was around $\bar{d}$ = 0.50 in favor of feedback conditions. Overall, the research literature paints a positive picture of *Student‐Centered* learning.

#### 3.1.2 Less individualized and less adaptive teacher‐centered education (*Teacher‐Centered*)

There has been considerable research in *Teacher‐Centered* education as well over the years. In the 1960s, during the Lyndon Johnson administration in the United States, a massive experiment called *Project Follow Through* was initiated to test the efficacy of a range of instructional strategies. The intent was to evaluate the relative advantages of models of instruction that ranged from Direct Instruction (i.e., DISTAR) to so‐called Open Education (i.e., based on the British Infant School Model). After years of testing and millions of dollars spent, only one really striking finding emerged: That *direct instruction* advantaged learners in terms of both measures of achievement and affect, outperforming other models by as much as 1.5 *SD* (standard deviation). While a great deal of controversy surrounds the conduct and findings of this large‐scale educational trial, its results set a tone of teacher‐centeredness that is still influential (Magliaro, Lockee, & Burton, 2005).

The most recent addition to the direct instruction literature comes from Stockard et al. (2018). The report, published in *Review of Educational Research*, entitled “The Effectiveness of Direct Instruction Curricula: A Meta‐Analysis of a Half‐Century of Research,” synthesized 328 studies involving 413 study designs and almost 4,000 effect sizes. Effect sizes, calculated between the Direct Instruction group and a comparison group (not specifically described), were reported for Reading ($\bar{d}$ = 0.51), math ($\bar{d}$ = 0.55), language ($\bar{d}$ = 0.54), and spelling ($\bar{d}$ = 0.66), interpreted as medium effect sizes by Cohen's (1988) criteria. This report suggests that teaching basic skills and competencies through a Direct Instruction curriculum is at least as effective as the best forms of individualized and adaptive instructional systems and approaches. This quote from the Stockard et al. report summarizes their view as to the distinction between *Teacher‐Centered* and *Student‐Centered* instruction:

Direct Instruction builds on the assumption that all students can learn with well‐designed instruction. When a student does not learn, it does not mean that something is wrong with the student but, instead, that something is wrong with the instruction. Thus, the theory underlying DI lies in opposition to developmental approaches, constructivism, and theories of learning styles, which assume that students’ ability to learn depends on their developmental stage, their ability to construct or derive understandings, or their own unique approach to learning. Instead, DI assumes all students can learn new material when (a) they have mastered prerequisite knowledge and skills and (b) the instruction is unambiguous. (p. 480)

This quotation acknowledges that there is a marked distinction between *Teacher‐Centered* and *Student‐Centered* learning. Our premise is that the answer to the question regarding *Teacher‐Centered* and *Student‐Centered* classrooms is not *either/or* but a spectrum of practices that usually avoids either extreme. The central question posed in this systematic review is the location of the sweet spot on the *Teacher‐Centered*/*Student‐Centered* spectrum. Where and when should the teacher maintain control of the sort described by Stockard et al. (2018) and where and when can students take more ownership of their own leaning processes?

#### 3.1.3 Comparing teacher‐centered and student‐centered instructional practices

A large‐scale examination (Hattie, 2008) of variables relating to various influences on educational outcomes of both *Teacher‐Centered* and *Student‐Centered* offers an opportunity to examine instructional practices side‐by‐side (See Table 1). Second‐order meta‐analyses relating to the teacher, the school, the curriculum, the home, etc. found average effect sizes for a number of instructional approaches that are shown in Table 1. Some of these approaches are clearly teacher‐centered, while some are more learner‐centered, and some have elements of both (or can be either depending on their application). Judging from these results, it is difficult to establish a clear pattern; *Student‐Centered*, *Teacher‐Centered* and both/either can be highly effective or not so effective. Clearly, a more in‐depth analysis is called for (Table 2).

**Table 1:** Results of second‐order meta‐analyses of selected educational practices (ordered by average effect size)

Instructional/pedagogical approach	Activity category (*Teacher‐Centered, Student‐Centered or both/either*)	Average effect size
Reciprocal teaching	Both/either	+0.74
Feedback to students	Both/either	+0.73
Problem‐solving teaching	Both/either	+0.61
Cooperative versus individualistic learning	Both/either	+0.59
Direct instruction	Teacher‐centered	+0.59
Peer tutoring	Learner‐centered	+0.55
Cooperative versus competitive learning	Both/either	+0.54
Cooperative learning versus other strategies	Both/either	+0.41
Inductive teaching	Teacher‐centered	+0.33
Inquiry‐based teaching	Both/either	+0.31
Problem‐based learning	Both/either	+0.15
Learner control of learning	Learner‐centered	+0.04
Open versus traditional education	Learner‐centered	+0.01

*Note*: Based on Hattie, (2008). *Visible learning: A synthesis of over 800 meta‐analyses related to achievement*. London: Routledge.

**Table 2:** Duvall & Tweedie's Trim and Fill

	*k*	$\bar{g}$	Lower 95th	Upper 95th	*Q* value
Observed values	365	0.44	0.38	0.50	3095.89
Adjusted values	0	0.44	0.38	0.50	3095.89

### 3.2 The pragmatics of teaching and learning

One might be tempted to organize some of these practices according to a spectrum of more and less constructivist practice. However, since constructivism has many different strands, both philosophically and pedagogically (Phillips, 1995), and since those strands vary significantly and counter‐intuitively in the degree of teacher‐centeredness they tend to imply, other approaches organize teaching practice more directly. These approaches label instructional strategies from more *Student‐Centered* (e.g., collaborative learning, discovery learning, problem‐based learning, inquiry‐based learning) to more teacher‐centered (e.g., direct or explicit instruction, didactic and expository instruction, lecturing, lecture‐discussion, drill, and practice).

#### 3.2.1 The genesis of this project

The current project deconstructs teaching and learning according to the events (or dimensions) associated with instructional conditions. Any of these events can be either more *Teacher‐Centered* or *Student‐Centered*. A more *Teacher‐Centered* environment is one where teachers are in charge of most of the instructional events. A more *Student‐Centered* classroom is one in which teachers pass on control over the responsibility for many of the instructional events to learners, thereby acting as guides rather than directors. These events are then isolated and rated, and a composite can be constructed that will yield a greater‐to‐lesser *Student‐Centered* scale along a continuum of instructional practices. This approach is multidimensional and avoids problems associated with the vague and somewhat confusing nature the first approach (i.e., holistically, more constructivist vs. less constructivist) and the inexact labeling (i.e., inquiry learning) of the second. It also has the advantage of allowing for the examination of clusters or combinations of instructional events that will be more practically relevant to k‐12 education.

There is support for this approach in the conclusion of Gersten et al. (2008), who was tasked with conducting a meta‐analysis of mathematics teaching practices of *Teacher‐Centered* and *Student‐Centered* classroom. They noted: “The Task Group found no examples of studies in which learners were teaching themselves or each other without any teacher guidance; nor did the Task Group find studies in which teachers conveyed … content directly to learners without any attention to their understanding or response. The fact that these terms, in practice, are neither clearly nor uniformly defined, nor are they true opposites, complicates the challenge of providing a review and synthesis of the literature…” (p. 12). Similarly, the National Mathematics Advisory Panel Final Report (2008) noted that most teachers do not rely on one single methodology (i.e., either/or, the extremes of teacher‐directedness or learner‐centeredness) but attempt to blend the two so that each is strengthened by the other.

In an attempt to help settle the issue in regards to inquiry instruction (in particular) versus direct instruction in k‐12 education, a team of researchers (Cobern et al., 2010), funded through NSF/IERI, conducted a 4‐year set of large‐scale RCTs comparing inquiry methods of teaching (*Student‐Centered*) with direct instruction (*Teacher‐Centered*). Results suggested that both models produced significant pretest‐posttest learning, but that there was no significant difference between the classroom models. One of their conclusions was that “… soundly constructed lessons, involving learner engagement, and competently taught by good teachers, are as important for development as to whether a lesson is cast as inquiry or direct instruction. Thus, the promotion of one mode of instruction over the other, where both are based on sound models of expert instruction, cannot be based simply on content acquisition alone” (p. 37). This result runs counter to the findings of a meta‐analysis by Schroeder, Scott, Tolson, Huang, and Lee (2007) of instructional practices in science education, where an average effect size of $\bar{d}$ = 0.65 was found for *Inquiry Strategies*, but in the write‐up of this review it is unclear what served as the control condition.

While it seems quite clear that both *Student‐Centered and Teacher‐Centered* instructional practices can contribute to learning, it is not at all clear how they work together and in what instructional domains. This project seeks answers to these questions.

### 3.3 Description of the intervention

The main research question of this meta‐analysis is: Can more *Student‐Centered* (i.e., more adaptive and individualized) approaches to k‐12 instruction be distinguished from more *Teacher‐Centered* approaches, and if they can, what approaches work best in terms of their effect on student achievements and what substantive (including combinations among dimensions) and demographic factors moderate these effects?

A concrete example of the advantage of using this approach compared to other classification schemes can be observed in the literature of cooperative and collaborative learning. While cooperative learning strategies tend to be more *Teacher‐Centered*, and collaborative learning tends to be more *Student‐Centered* (i.e., there tend to be more rules for delivering cooperative learning than there are in collaborative learning), great variance can be observed in the way the steps (e.g., group composition, task selection, role assignment, assessment methods) in each are operationalized. As a teaching/learning strategy, cooperative learning is perhaps the most heavily researched and best‐understood technique for involving learners in small‐group, process‐oriented learning. However, it can be viewed as either *Teacher‐Centered* or *Student‐Centered* depending on how its components are implemented.

For better understanding and more successful practical application, educational practices subsumed under this generic pedagogical idea of adaptive teaching and individualized learning deserves a valid conceptual working model, both inclusive enough to account for various forms of personalized/individualized instruction and sufficiently sensitive to fluctuations due not only to the influence of numerous moderator variables, but also to nuanced qualities of particular instructional approaches themselves. *Student‐Centered* instructional strategies could, in our view, serve such an overarching conceptual framework with adequate explanatory power, but only if operationalized properly to avoid an oversimplified dichotomy such as inductive versus deductive or constructivist versus direct instruction.

### 3.4 How the intervention might work

The phenomenon being investigated in this review is not an *intervention* in the normal way that this word is used in the experimental literature. It is more correctly a set of instructional practices that have defined along a continuum from extremely *Teacher‐Centered* (where the teacher is the boss in control of all instructional events) to extremely *Student‐Centered* (i.e., where the teacher is a guide and facilitator, even sometimes an equal partner). As such, any classroom research, regardless of the *intervention* being investigated, is eligible so long as there is sufficient detail provided as to what each group did.

Following a review of the literature by the research team on instructional practices in grades k‐12, we developed a list of instructional events (or dimensions) that can be rated on a *Teacher‐Centered* to *Student‐Centered* continuum. These are: (a) Flexibility in Course Planning—degree to which teachers/learners participate in course design, setting objectives, selecting or creating materials; (b) Pacing of Instruction/Learning—degree to which teachers/learners set the pace and content navigation of learning activities; (c) Teacher's Role—degree to which the teacher's role in the classroom ranges from lecturer to lecturer/authority figure/facilitator/ guide/partner; and (d) Adaptability of Instruction—degree to which materials and activities are generic or modified for individual students.

To define the key qualities of instruction as *adaptive* and *individualized*, (referred to here as *Student‐Centered*) for the purposes of this systematic review, we have deconstructed teaching and learning according to the events associated with them. Accordingly, a more *Student‐Centered* (more adaptive and individualized) classroom is one in which students play a more central role in the conduct of the instructional events. Conversely, if teachers dominate the instructional events, the classroom might be referred to as *less adaptive*. We have isolated these categories of instruction in reports of primary classroom research and rated them individually on a *Teacher‐Centered* to *Student‐Centered* continuum. Each event could then be: (a) Examined separately to determine their individual strengths; (b) examined in clusters as combinations of events; or (c) collapsed into a multidimensional composite that would yield a *greater‐to‐lesser* distinction between two different instructional settings. This approach avoids problems associated with either subjectively defining instructional conditions as *Teacher‐Centered* versus *Student‐Centered* or the specific labeling of them, as, for instance, PSI, mastery learning, etc. It also has the advantage of allowing us to examine instructional events in isolation and in various combinations in the search for optimal instructional practices.

### 3.5 Why it is important to do the review

Most of the significant effects from the meta‐analyses described in the first section of this report on the topic cluster around $\bar{d}$ = 0.40, but the data also reflect a wide range of effects depending on the whole spectrum of moderator variables. Also, the overall picture painted by these meta‐analyses is less useful today as most are now dated. Of special concern to us is the fact that both earlier and recent meta‐analyses are rather limited in scope and focus of interest, addressing very specific instructional practices. There were no serious attempts to find and conceptualize pedagogical commonalities among the interventions in question that would allow treating them within the same class of phenomena broadly depicted as individualized learning and adaptive teaching. Thus, a review that is broad in scope and summarizes up to‐date‐evidence is a next logical step in investigating these phenomena.

### 3.6 Objectives

The main objective of this review is to summarize research on the effectiveness (in terms of learning achievement outcomes) of adaptive and individualized instructional interventions operationally defined here as more *Student‐Centered* pedagogical approaches. The overall weighted average effect size will be an indication of that. Additionally, and no less important, the review aims to provide a better understanding of what circumstances (e.g., with what populations of learners, for what subject matters) the effects of adaptive and individualized instruction reach their highest potential, and what conditions may depress them. To explore this, a set of substantive and demographic study features are coded and subjected to moderator variable analyses.

The outcomes of this review will inform education practitioners and the research community of the best instructional practices, preconditions for their successful implementation, and potential pitfalls, as well as directions for further empirical research in the area.

## 4 METHODS

### 4.1 Criteria for considering studies for this review

#### 4.1.1 Types of studies

Only studies that considered the difference between the two groups were eligible for inclusion in this review. Included are studies that are experimental (i.e., Randomized Control Trials) or high‐quality Quasi‐Experimental Designs (i.e., statistically verified group equivalence or adjustment) in design that adequately addressed the research question of group comparisons, contained legitimate measures of academic achievement (i.e., teacher/researcher‐made, standardized), and reported sufficient statistical information for effect size extraction.

#### 4.1.2 Types of participants

The participants are students in k‐12 formal educational settings (~ages 5–18) eventually leading to a certificate, diploma, degree, or promotion to a higher level. Educational interventions take place either in the classroom, via distance education, or as a blended intervention (various combinations of classroom and distance education).

#### 4.1.3 Types of interventions

As described earlier, the intervention in question (an experimental condition) was considered to be any combination of instructional events that is rated higher in *Student‐Centered* qualities than a comparison (control) condition. Student participation in decisions about or control over the selection of study materials and learning activities, pacing of instruction, adapting learning for students’ individual needs, interests, backgrounds, etc., as well as various degrees of involvement in “partnership” with teachers, constitute, in our view, such *Student‐Centered* qualities of instruction. Two experienced independent reviewers coded instructional conditions featured in a given primary study (on a scale from 1–5) to reflect the extent to which each group possessed these qualities. Below we describe dimensions that were in the focus of our review.

Within each eligible comparative study all participation groups were coded for the four effect dimensions using a five‐point scale, as follows:

- Dimension of *Teacher's Role* represents a continuum of a teacher's major responsibilities for organizing/delivering instruction/managing classroom activities, etc.

**Coding:** Describes the teacher's predominant role in the teaching/learning process:

1. Teacher almost exclusively lectures, is the main source of content‐relevant information and/or an authority figure.
2. Teacher provides some guidance, feedback, initiates and supports discussions, etc.
3. Teacher functions as a guide, coach, tutor, provocateur of thinking.
4. Teacher functions as a colleague, partner in learning.
5. Teacher almost exclusively acts as a facilitator of learning, responding to students’ specific needs (follows students’ lead, consults, clarifies, encourages, etc.).

- Dimension of *Pacing* reflects the degree of student control over the time of instruction/learning and over the progression through the course content (i.e., pedagogical flexibility—revisiting/selecting/skipping/reordering topics and tasks).

**Coding:** Describes the degree to which students are given control over course progression:

1. Instruction is highly structured and progresses step‐by‐step; no flexibility is allowed.
2. Minor degree of either logistical or pedagogical flexibility is available to students.
3. Program/teacher's control over course progression is balanced with that of students.
4. Students have a substantial amount of flexibility in course progression.
5. High degree of flexibility (up to the point of completely self‐paced and/or self‐planned/self‐managed learning).

- Dimension of *Flexibility* describes the degree of student control over course design, selection, and the provision of study materials and the setting up of learning objectives.

**Coding:** Describes the degree to which teachers/students participate in course planning:

1. No involvement of students (most is determined by the teacher or program/curriculum).
2. Student involvement in at least one of the components of course planning is present but limited.
3. Teachers and students collaborate in the course planning, but teacher's role is still dominant.
4. Teachers and students collaborate in the course planning equally.
5. High student involvement—students play a leading role in course planning and selection of learning materials.

- Dimension of *Adaptability of Instruction* describes the degree to which levels or modifications in instructional process is provided to accommodate individual students.

**Coding:** Describes the degree to which instruction takes into account students’ needs/interests/level of knowledge:

1. Learning materials, settings, group formation (if any), activities and other work arrangements are predetermined and unchanged throughout the instruction (e.g., standardized or required curriculum).
2. Minor modifications are allowed to either learning materials, group composition, or the context of instruction.
3. Elements of either individualized feedback, or role and tasks assignments based on students’ interests and/or previous achievements, etc.
4. Adapting several instructional components (in combinations) to students’ individual needs/interests/levels of knowledge.
5. High levels of joint Adaptability of several components of instruction.

Based on the results of this coding (implemented independently by two reviewers compared and finalized in discussions), numeric values for each participating group were derived. The sum of these values determined the experimental (higher total) and control (lower total) conditions in every included study. The differential score was subsequently calculated to reflect the degree of student‐centered (*Student‐Centered*) components of instruction and to serve as a “continuous” predictor in meta‐regression of effect sizes against the “strength” of the intervention. In the Results section this variable is depicted as “*Student‐Centered* Total Differential Score” (i.e., sum of scores for the experimental group minus sum of scores for the control group) with a theoretical range from 1 (one point difference in coding on a single dimension) to 16 (maximum difference between groups on all four coded dimensions).

Similarly, we determined and reflected the number of dimensions with differential scores higher than zero (i.e., on how many dimensions adaptive qualities of the instruction were present in the experimental group to a greater extent than in the control group). This variable is labeled “Difference by Dimension” and could range from 1 (difference on a single dimension) to 4 (difference on all four dimensions), regardless of the magnitude of that difference.

Finally, we wanted to trace and analyze the source of the difference. To that end, a categorical variable *Source of the Difference* was designed to reflect what dimensions in what combinations contributed to the magnitude of the respective effect size. Initial letters of each of the coded dimension depicted levels of this variable. For example, F_T_A stands for some difference on the dimensions of *Flexibility*, *Teacher's Role*, and *Adaptability of Instruction*, with zero differential score on the dimension of *Pacing*.

Decisions about the completeness of the reported information were made at three points in time. First, two independent coders decided in general on each study's inclusion/exclusion status (overall qualitative judgment). Second, when dimensions were actually coded, reviewers searched for all relevant information in the study and if this information was not found, assigned the valued of “999” (or missing information) subsequently excluding studies with more than one “999.” Third, at the analysis stage studies with “999” were converted into zeroes, indicating no difference between the two respective conditions, and if after this transformation the overall composite score was zero these studies were also excluded.

As a result, only studies judged by coders to have provided sufficient description were retained for analysis.

#### 4.1.4 Types of outcome measures

##### Primary outcome

All types of objective measures of academic achievements were considered. Their psychometric features (e.g., standardized, nonstandardized teacher/researcher‐made assessment tools) and type of representativeness (e.g., cumulative final examinations or averages of several performance tasks covering various components of the course/unit content) were documented and used in subsequent moderator variable analyses. Self‐assessments were excluded, as well as attitudinal and behavioral measures.

#### 4.1.5 Duration of follow‐up

To maximize coverage of primary research, fully compatible in terms of outcome measures, only immediate post‐test results were considered. Various forms of delayed post‐tests were documented and their time lags categorized to inform further reviews.

#### 4.1.6 Types of settings

As stated earlier, k‐12 formal educational settings (~ages 5–18), in educational programs leading to advancement to the next academic level/grade, were required in the current meta‐analysis. Other settings (i.e., homeschooling, auxiliary programs, summer camps, vocational workshops, etc.) were excluded.

### 4.2 Search methods for identification of studies

#### 4.2.1 Electronic searches

Following the *Guidelines of the Campbell Collaboration* (Kugley et al., 2017), in order to retrieve a broad base of studies to review we started by having an experienced Information Specialist search across an array of bibliographic databases, both in the subject area and in related disciplines. The following databases were searched for relevant publications: ABI/Inform Global (ProQuest), Academic Search Complete (EBSCO), ERIC (EBSCO), PsycINFO (EBSCO), CBCA Education (ProQuest), Education Source (EBSCO), Web of Knowledge, Engineering Village, Francis, ProQuest Dissertations & Theses Global, ProQuest Education Database, Linguistics and Language Behavior Abstracts (ProQuest).

The search strategy was tailored to the features of each database, making use of database‐specific controlled vocabulary and search filters. Searches were limited to the year 2000–2017, and targeted a k‐12 population. The following is an example from the ERIC database:

(AB (adaptive OR personalized OR personalized OR individuali*) AND AB (pedagog* OR learning OR teaching OR instruction OR education OR classroom OR curriculum)

OR (AB “self direct*” OR “self regulate*”) OR (DE “Open Education” OR DE “Discovery Learning” OR DE “Individual Activities” OR DE “Student‐Centered Curriculum” OR DE “Student Centered Learning” OR DE “Mastery Learning” OR DE “Independent Reading” OR DE “Independent Study” OR DE “Individualized Instruction” OR DE “Competency‐Based Education” OR DE “Individual Instruction” OR DE “Individualized Programs” OR DE “Individualized Reading” OR DE “Individualized Transition Plans” OR DE “Learner Controlled Instruction” OR DE “Pacing” OR DE “Individual Testing” OR DE “Adaptive Testing” OR DE “Experiential Learning” OR DE “Learner Engagement” OR DE “Cooperative Learning”))

AND

(DE “Program Validation” OR DE “Academic Achievement” OR DE “Instructional Improvement” OR DE “Progress Monitoring” OR DE “Educational Assessment” OR DE “Instructional Effectiveness” OR DE “Program Evaluation” OR DE “School Effectiveness” OR DE “Evidence” OR DE “Outcomes of Education” OR DE “Program Effectiveness”)

AND

(DE “Pretesting” OR DE “Pretests Posttests” OR DE “Control Groups” OR DE “Experimental Groups” OR DE “Matched Groups” OR DE “Mixed Methods Research” OR DE “Randomized Controlled Trials” OR DE “Effect Size” OR DE “Quasiexperimental Design” OR DE “Comparative Analysis”)

Limiters—Date Published: 20000101‐20171231; Educational Level: Elementary Education, Grade 1, Grade 2, Grade 3, Grade 4, Grade 5, Grade 6, Grade 7, Grade 8, Grade 9, Grade 10, Grade 11, Grade 12, High Schools, Junior High Schools, Kindergarten, Middle Schools, Primary Education, Secondary Education; Publication Type: Books, Collected Works (All), Dissertations/Theses (All), ERIC Publications, Information Analyses, Journal Articles, Numerical/Quantitative Data, Reports—Descriptive, Reports—Evaluative Reports, Reports—Research.

#### 4.2.2 Searching other resources

##### Theses/Conference papers/Research reports

Database searching was supplemented by using the Google search engine to locate additional articles, but principally grey literature (research reports, conference papers, theses, and research published outside conventional journals). Finally, an in‐house database of empirical studies of teaching methods, assembled by the research team from previous research reviews, was searched and produced an additional 254 studies. While these studies had been previously collected, the same set of inclusion criteria used for other studies were applied to them.

### 4.3 Data collection and analysis

#### 4.3.1 Selection of studies

The overall set of inclusion/exclusion criteria for the meta‐analysis contained the following requirements:

- Be publicly available (or archived) and encompass studies from 2000 to the present.
- Feature at least two groups of different instructional strategies/practices that can be compared according to the research question as *Student‐Centered* and *Teacher‐Centered* instruction.
- Include course content and outcome measures that are compatible with the groups that form these comparisons.
- Contain sufficient descriptions of major instructional events in both instructional conditions.
- Satisfy the requirements of either experimental or high‐quality quasi‐experimental design.
- Is conducted in formal k‐12 educational settings eventually leading to a certificate, diploma, degree, or promotion to a higher grade level.
- Contain measures representative of course achievement (i.e., teacher/researcher‐made, standardized).
- Contain sufficient statistical information for effect size extraction.

#### 4.3.2 Data extraction and management

Two researchers independently conducted abstract screening and full‐text review of studies identified through the whole complex of searching activities, compared notes, discussed and resolved disagreements, and documented reliability rates. Similar procedures were employed for effect size extraction and coding of moderator variables.

#### 4.3.3 Effect size extraction and calculation

One of the selection criteria was “Contain sufficient statistical information for effect size extraction,” so that an effect size could be calculated for each independent comparison. This information could take several forms (in all cases sample size data were required):

- Means and standard deviations for each treatment and control group;
- Exact *t* value, *F*‐value, with an indication of the ± direction of the effect;
- Exact *p* value (e.g., *p* = .011), with an indication of the ± direction of the effect;
- Effect sizes converted from correlations or log odds ratios;
- Estimates of the mean difference (e.g., adjusted means, regression β weight, gain score means when *r* is unknown)
- Estimates of the pooled standard deviation (e.g., gain score standard deviation, one‐way *ANOVA* with three or more groups);
- Estimates based on a probability of a significant *t* test using α (e.g., *p* < .05); and
- Approximations based on dichotomous data (e.g., percentages of students who succeeded or failed the course requirements).

Effect sizes were initially calculated as Cohen's *d* and then converted to Hedges’*g* (i.e., correction for small samples). Standard errors ($SE_{d}$) were calculated for $\bar{d}$ and then converted to standard errors of *SE*_*g*_ applying the correction formula for *g*. Hedges’ *g*, *SE*_*g*_, and sample sizes (i.e., treatment and control) were entered into *Comprehensive Meta‐Analysis* 3.3.07 (Borenstein et al., 2014) where statistical analyses were performed.

The effect sizes were coded for precision of calculations and analyzed in moderator variable analysis.

#### 4.3.4 Description of methods used in primary research

True experimental and quasi‐experimental studies were included as far as they feature two educational interventions covering the same content (required knowledge acquisition and/or skill development). They were assessed on compatible outcome measures, where one group (experimental) is greater in *Student‐Centered* qualities (as described earlier) compared to the other (control) group with fewer *Student‐Centered* qualities. Reporting quantitative data sufficient for an effect size extraction was a necessary condition for study inclusion.

#### 4.3.5 Criteria for determination of independent findings

There are several potential major threats to the independence of the findings. These are: (a) Repeated use of data coming from the same participants (i.e., dependence); (b) reporting multiple outcomes of the same type; and (c) aggregating outcomes of different types representing the same sample of participants (does not apply to this review, as it is limited to learning achievement outcomes only). The means that we used for ensuring data independence were that no group of participants was used more than once, resulting in most cases in only one effect size per study; and only one outcome measure was used in each comparison (either cumulative or composite achievement score).

#### 4.3.6 Details of study coding categories

In addition to the coding dimensions of *Student‐Centered* pedagogical qualities that would determine proper comparisons for effect size extraction, the following groups of study coding categories were used in the review. First, study methodological quality was assessed for features such as design type, the fidelity of treatment implementation, attrition, and the unit of assignment/analysis (Cooper, Hedges, & Valentine, 2009). Within the same category, we coded for outcome source and psychometric quality of the assessment tools, as well as for the precision of procedures used for effect size extraction and for equivalence of instructor and study materials. Jointly, these methodological study features were used in moderator variable analyses to inform us of any potential threats to all types of study validity (Cooper et al., 2009).

Substantive study features further clarify descriptions of *Student‐Centered* pedagogical qualities by specifying theoretical models underlying instructional practices under review, treatment duration, instructor's experience, provision of professional development for teachers, and training for students, whenever it is required by specific instructional intervention. Demographic study features encompass learners’ age, educational background, and ability level, as well as, subject matter studied. All of these study features were subsequently analyzed as moderators for their potential impact on treatment effects.

#### 4.3.7 Assessment of risk of bias in included studies

Assessment of the risk of bias was accomplished in several ways:

#### 4.3.8 Sensitivity analysis

Sensitivity analysis was performed to determine if issues such as research design, effect size extraction methods, instructor and material equivalence, publication bias, and assessment tool category, might have introduced bias into the results. It also involves a “one study removed” analysis of the distribution effect sizes. For the results of this analysis please see Table 5a–e.

#### 4.3.9 Assessment of reporting biases

Publication bias was assessed based on the examination of a Forest Plot and associated “Trim and Fill” analysis plus other tests such as Classic Fail‐safe analysis and Orwin's Fail‐safe *N* (Orwin, 1983).

#### 4.3.10 Assessment of heterogeneity

Homogeneity assessment, sometimes called an analysis of precision, was accomplished using the fixed model of analysis. The following indicators are reported and discussed:

Q‐Total, df, test of the null hypothesis, *I* ^2^ (percentage of error variance over and above chance), and tau^2^ (average variability used in the calculation of random weights).

#### 4.3.11 Data synthesis

- Data are synthesized, initially, under the random effects model, and includes the following statistics: Overall weighted random effects analysis with the statistics of $\bar{g}$, *SE*_*g*_, *V*_g_, upper and lower limits of the 95th confidence interval, *z*_*g*_, and *p* value;
- Heterogeneity is estimated using *Q*‐Total, *df*, and *p* value. *I* ^2^ (i.e., percentage of error variation) and tau^2^ (i.e., average heterogeneity) is also calculated and reported.
- Meta‐regression (single and multiple) is used to determine the relationship between covariates and effect sizes; and
- Mixed‐model (i.e., random and fixed) moderator variable analysis is used to compare categories of each coded moderator variable. *Q‐*Between, *df*, and *p* value are used to make decisions about the significance of difference among levels of each categorical variable.

The protocol for this review was published in the Campbell Library, August 2016: https://campbellcollaboration.org/media/k2/attachments/Bernard_Operationalized_Adaptive_Teaching_Title.pdf

## 5 RESULTS

### 5.1 Description of studies

#### 5.1.1 Results of the searches

**Figure 1:**
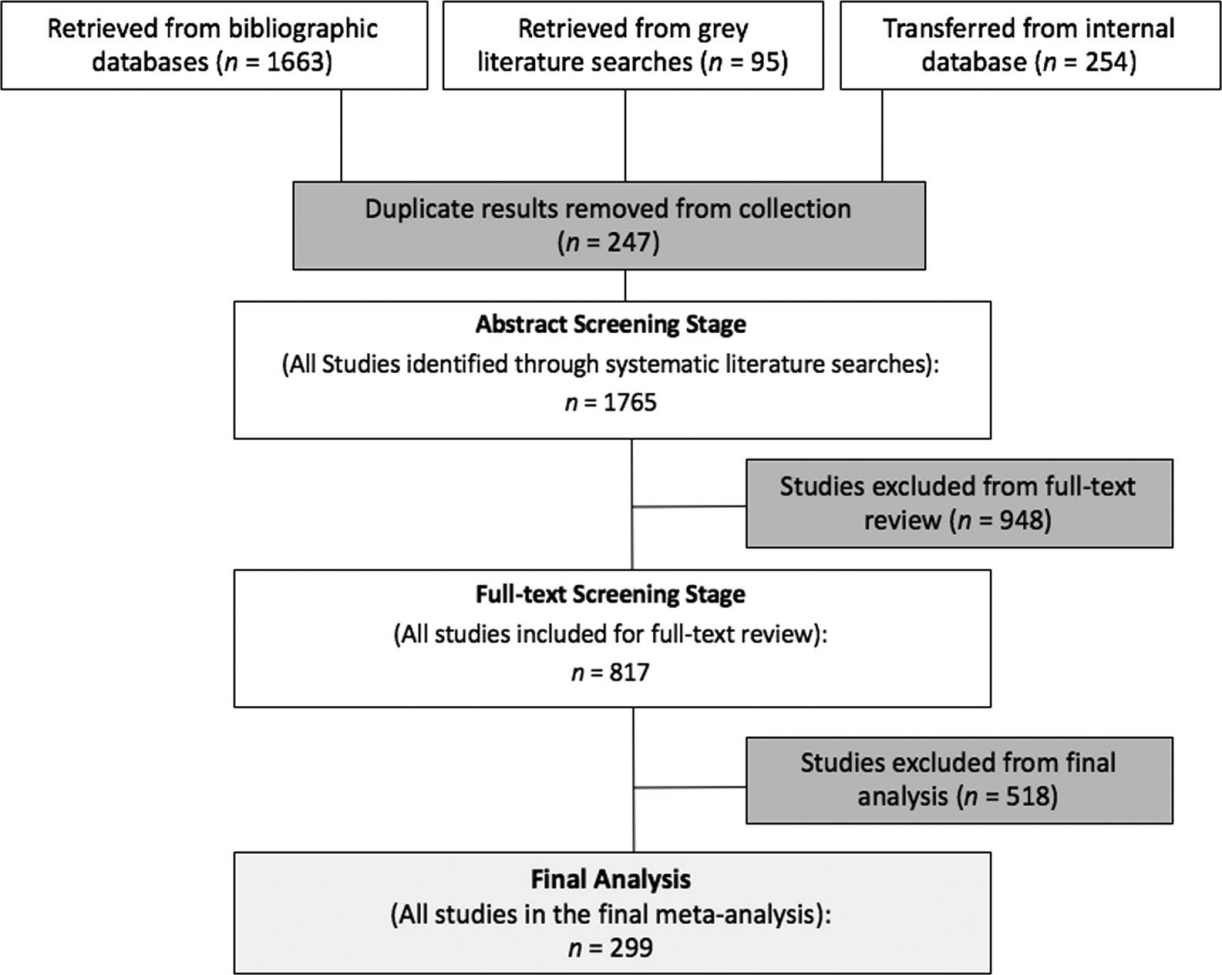
Flow diagram of the review process

All searches were conducted by a fulltime Information Specialist (MLS level) and member of the Systematic Review Team at the Centre for the Study of Learning and Performance at Concordia University in Montreal, QC, Canada. As shown in the PISA flowchart in Figure 1, there were three sources of studies: (a) 1,663 studies from dedicated bibliographic searches detailed in the Method; (b) 95 studies retrieved from the grey literature; and (c) 254 studies transferred from an internal database of studies retrieved for a larger meta‐analysis that includes all grade levels, but with the same inclusion/exclusion criteria as the current study. Figure 1 details the results at each stage of the search and retrieval process. All bibliographic information was exported into an Endnote database and managed from there.

**Figure 2:**
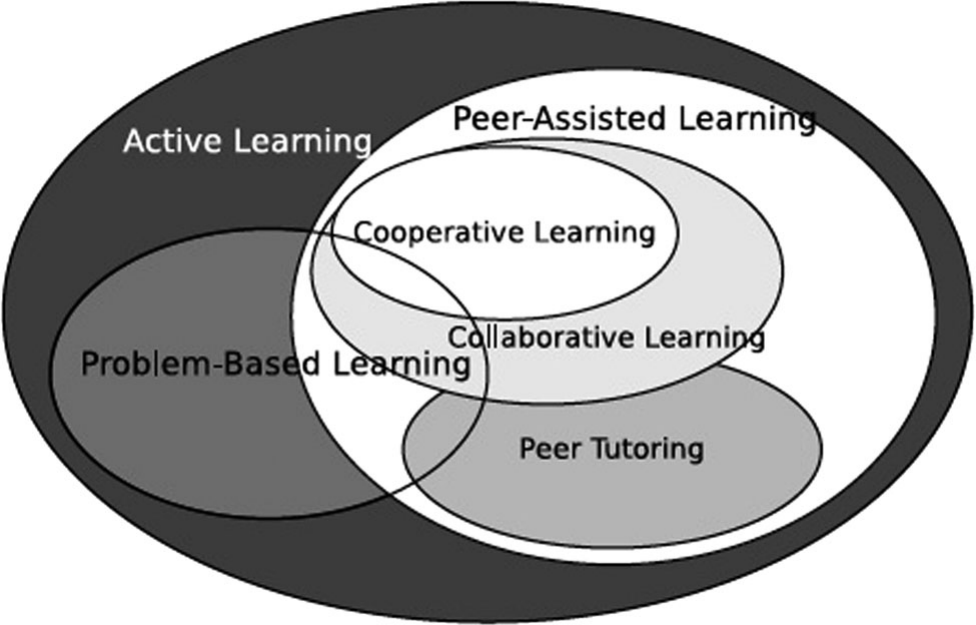
Venn diagram of the overlap among methods of active learning (Student‐Centered; Bishop & Verleger, 2013, p. 6)

Duplicate studies were removed (*n* = 247) and the remaining 1,765 studies were subjected to an abstract screening process. In all, 817 studies were retrieved as full‐text documents. Examination of these studies proceeded according to the details described in the Method. A total of 518 full‐text documents were excluded for reasons detailed in the inclusion/exclusion description in the Method, leaving 299 studies that were included in the final analysis. In the final stage, 365 independent effect sizes were extracted from these studies, coded, and analyzed.

#### 5.1.2 Included studies

There are 365 effect sizes (299 individual studies) included in this review, representing 43,175 treatment and control participants. References to these 299 studies appear in the section entitled *References to included studies.* Please see Table S13 for complete statistical information for the 365 effect sizes.

#### 5.1.3 Excluded studies

A total of 1,613 studies were excluded from this review. Figure 1 shows how this number diminished over the course of the review and selection process and references to these excluded studies are presented in the section entitled *References to excluded studies* (found in Online Supplement 1).

### 5.2 Risk of bias in included studies

In assessing the quality of included studies we used the following criteria: Methodological quality moderators, publication and sensitivity bias analysis, data independence, and sufficiency of the description of instructional practices.

#### 5.2.1 Publication bias analysis

Borenstein et al. (2014) state:

“The basic issue of publication bias is that not all completed studies are published, and the selection process is not random (hence the “bias”). Rather, studies that report relatively large treatment effects are more likely to be submitted and/or accepted for publication than studies [that] report more modest treatment effects. Since the treatment effect estimated from a biased collection of studies would tend to overestimate the true treatment effect, it is important to assess the likely extent of the bias, and its potential impact on the conclusions” (Publication Bias Report, Comprehensive Meta‐Analysis, 2014).

Thus, this report includes an extensive investigation of publication bias, as a potential source of difficulty and error in interpreting these results.

##### Funnel Plot analysis and Trim and Fill

**Figure 3:**
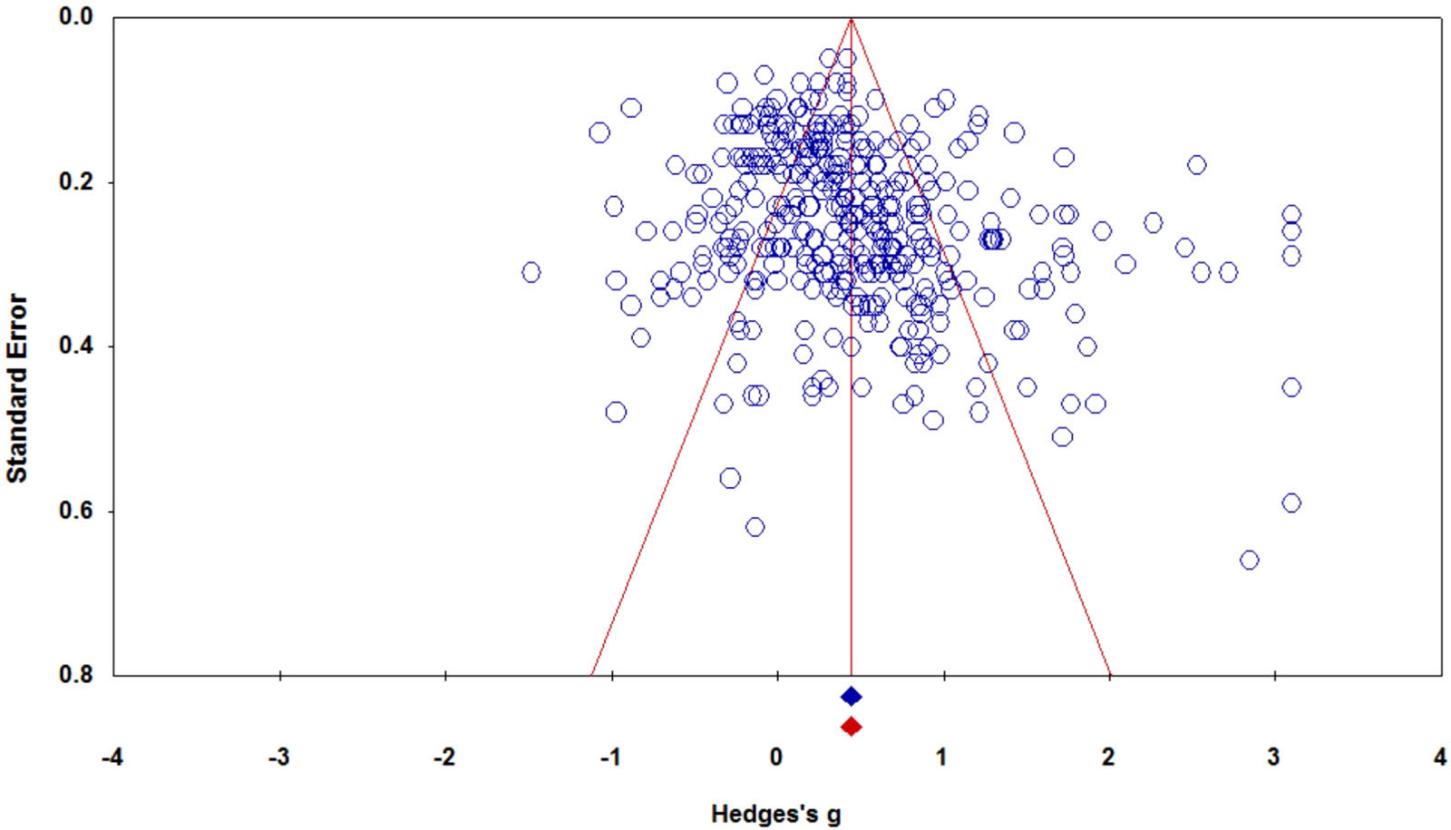
Random effects funnel plot (effect size by standard error). [Color figure can be viewed at wileyonlinelibrary.com]

A Funnel Plot (See Figure 3) and associated Trim and Fill procedure (Duval & Tweedie, 2000) of 365 effect sizes (See Table 3) indicate that there is no discernable publication bias on the negative side of the plot (i.e., left of the mean effect size) under the random effects model.

**Table 3:** Analysis of publication source

Categories	*k*	$\bar{g}$	*SE*	Lower 95th	Upper 95th	*z*‐value	*p* value	*Q*‐B	*df*	*p* value
Journal articles	302	0.46	0.03	0.40	0.53	13.69	.00			
Theses	8	0.31	0.12	0.08	0.54	2.60	.01			
Other	54	0.35	0.07	0.21	0.50	4.85	.00			
Total between								3.12	2	.21

Another indicator, Classic fail‐safe *N*, suggests that 121,993 additional effect sizes would be needed to bring the observed *p* value below alpha = .05 (i.e., 860 additional “null” effect sizes per each observed effect size). Also, Orwin's fail‐safe *N* (Orwin, 1983), suggests that 125 additional “null” effect sizes would be needed to bring the observed average effect size to a trivial level of $\bar{g}$= 0.10.

In addition, an analysis of publication type (See Table 3) indicated that journal articles ($\bar{g}$ = 0.46, *k* = 302), unpublished theses ($\bar{g}$ = 0.31, *k* = 8) and other unpublished documents (e.g., conference papers), ($\bar{g}$ = 0.35, *k* = 54) were not significantly different in mixed‐model moderator analysis (*Q*‐Between = 3.12, *df* = 2, *p *= .21). However, these nonsignificant findings should be viewed cautiously because of the small *k* for theses, possibly resulting from an issue of power.

Overall, there appear to be no serious issues of bias related to the analysis of published data.

#### 5.2.2 Sensitivity analysis

Sensitivity analysis examines issues in the data and coding that might affect the reliability of the results. First, we conducted a *one study removed* (Borenstein et al., 2014, CMA, Version 3.3.070) analysis of effect size and study sample on the variability of the individual data points across the distribution. Table 4 shows partial results of that analysis. The table contains six studies from the top of the distribution (highest effect sizes) and six studies from the bottom (lowest effect sizes, all negative). There is only a 0.01th difference in average effect size between the top and the bottom of the distribution when each study is removed sequentially. Also, the standard errors and the limits of the 95th confidence demonstrate the same consistency. Since the most problematic studies often reside on the peripheries of the distribution, large/small in effect size magnitude and large in sample size (i.e., high influence studies), the relative random weights were included in the last column. In the 12 studies displayed, their influence ranged from 0.13–0.28 on the upper end and 0.19–0.33 on the lower end indicating little concern for undue influence.

**Table 4:** Sample (top six studies and bottom six studies) of one study removed

Study names	Actual	One study removed				Relative wt.
	*g*	$\bar{g}$	*SE*	Lower 95th	Upper 95th	
1. Zohar_2 2008	3.10	0.44	0.03	0.38	0.50	0.20
2. Lamidi 2015	3.10	0.44	0.03	0.38	0.49	0.28
3. Garcâ_1 2006	3.10	0.44	0.03	0.38	0.50	0.26
4. Ben‐David 2009	3.10	0.44	0.03	0.38	0.49	0.27
5. Alfassi_1 2003	3.10	0.44	0.03	0.38	0.50	0.15
6. Alfassi_2 2003	2.85	0.44	0.03	0.38	0.50	0.13
360. Eysink_2 2009	−0.88	0.45	0.04	0.23	0.39	0.33
361. Furtak_1 2012	−0.97	0.45	0.04	0.23	0.39	0.19
362. Chang_2 2002	−0.97	0.45	0.04	0.23	0.39	0.25
363. Sola_2 2007	−0.98	0.45	0.04	0.23	0.39	0.29
364. Wesche 2002	−1.07	0.45	0.04	0.23	0.39	0.32
365. Bassett 2014	−1.48	0.45	0.04	0.23	0.39	0.25
Overall (*k* = 365)	0.44	0.44	0.03	0.38	0.50	100%

Several issue related to study design quality and methodology (See Table 5a–c) also suggest no or minimal potential bias in these results (data below reported according to the mixed model analyses):

- Bias in research design (Table 5a)—in moderator variable analysis, quasi‐experimental research designs versus randomized control trials, show no significant difference in average effect sizes ($\bar{g}$ = 0.46, *k* = 273 and $\bar{g}$ = 0.40, *k* = 90, respectively, *Q*‐between = 0.55, *df*  = 1, *p* = .46);
- Effect size extraction bias (Table 5b)—effects calculated from exact descriptive statistics (e.g., means and *SD*s, exact *t‐*values, exact *p* values) versus effects estimated based on other statistics with the element of some assumptions (e.g., reported nonexact significance levels, *p* < .05) indicates no observable bias ($\bar{g}$ = 0.37, *k *= 107 vs. $\bar{g}$ = 0.48, *k* = 257, respectively, *Q*‐between = 2.83, *df* = 1, *p* = .09).
- Bias in the quality of instrument types (Table 5c)—standardized tests versus modified or piloted standardized tests versus teacher/researcher made tests versus combinations of measure types indicates no bias in average effect sizes ($\bar{g}$ = 0.37, *k* = 78, versus $\bar{g}$ = 0.44. *k* = 79, versus $\bar{g}$ = 0.48, *k* = 196, vs. $\bar{g}$ = 0.35 vs. *k* = 11, respectively, *Q*‐between = 3.11, *df* = 3, *p* = .37).
- Bias in coding—Five sources of coding bias were recorded and are presented here as percentage of agreement and Cohen's Kappa (*κ*; i.e., inter‐rater reliability):

1. Abstract screening, 84.48% or *κ* = 0.69;
2. Full‐text review, 96.08% or *κ *= 0.92;
3. Decisions on number of effects per study, 92.78% or *κ* = 0.92;
4. Data extraction and effect size calculation, 96.21 or *κ* = 0.92; and
5. Study Feature coding (including the four primary dimensions), 92.23% or *κ *= 0.84.

**Table 5:** a–e Research design, extraction method, instrument type, and publication source

Codes	*k*	$\bar{g}$	*SE*	Lower 95th	Upper 95th	*z*‐value	*p* value	*Q*‐B	*df*	*p* value
**Research design—a**										
QED	273	0.46	0.03	0.39	0.53	13.18	.00			
RCT	90	0.40	0.07	0.28	0.53	6.17	.00			
Total between								0.55	1	.46
**Extraction method—b**										
Exact	107	0.37	0.05	0.27	0.47	7.22	.00			
Approximate	257	0.48	0.04	0.40	0.55	12.69	.00			
Total between								2.83	1	.09
**Instrument type—c**										
Standardized	78	0.37	0.00	0.28	0.46	7.95	.00			
Mod. Stand.	79	0.44	0.01	0.30	0.59	5.95	.00			
Teacher/Rcher.	196	0.48	0.00	0.39	0.57	10.26	.00			
Combo	11	0.35	0.02	0.10	0.60	2.78	.01			
Total between								3.11	3	.37
**Teacher class assignment—d**										
Same teacher	156	0.32	0.00	0.23	0.42	6.64	.00			
Diff. teacher	196	0.52	0.00	0.44	0.60	13.12	.00			
Total between								10.19	1	.00
**Teacher training—e**										
No	128	0.50	0.05	0.40	0.60	9.86	.00			
Yes	208	0.45	0.04	0.37	0.53	10.86	.01			
Total between								0.63	1	.43

*Note*: Missing data has been removed so *k* does not always equal 365.

Abbreviations: QED: quasi‐experimental design; RCT: randomized control trial.

These coding values are deemed to be within normal range and so no bias seems to be present.

Two other potential sources of bias that arose from classroom conditions were also tested:

- Bias in teacher assignment (Table 5d)—same teacher in both classrooms versus different teachers in each classroom ($\bar{g}$ = 0.32, *k* = 157 vs. $\bar{g}$ = 0.52, *k* = 196, *Q*‐between = 10.19, *df*  = 1, *p* = <.001. In this case, bias seems to be present, with different teachers in each classroom outperforming classrooms where the same teacher was assigned.
- Teacher training in *Student‐Centered* methods (Table 5e)—no teacher training versus teacher training ($\bar{g}$ = 0.50, *k* = 218 vs. $\bar{g}$ = 0.45, *k* = 208, *Q*‐between = 0.63, *df* = 1, *p *= .43). There appears to be no bias due to differential teacher training.

Overall, this assessment of methodological and classroom variables indicates only one area of concern: The same teacher assigned to both the treatment and control conditions or a different teacher assigned to each condition. Different teachers appear to produce significantly higher effect sizes than when the same teacher is used in the two conditions. While this form of bias is of concern by itself and was further explored in the subsequent analyses, in light of all of the other bias issues tested and found to be equivalent in their influence on the treatment effect, it is unlikely that this issue alone affected the ultimate conclusions of this review.

### 5.3 Synthesis of results

#### 5.3.1 Primary analysis

##### Basic question

The first question involved the overall average effect on achievement outcomes of more adaptive instruction as it is reflected in the difference between more *Student‐Centered* instructional conditions (the treatment condition) and less *Student‐Centered* conditions (the control condition). It is important to understand that this is not necessarily a contrast between *Student‐Centered* classrooms and *Teacher‐Centered* classrooms. It is instead the differential in ratings (on four effect size‐defining dimensions outlined in the Method section) between a treatment (more *Student‐Centered*) and control condition (less *Student‐Centered*) that range from equal (i.e., zero, treatment and control are equally *Student‐Centered* and *Teacher‐Centered*) to large, (i.e., up to +3 or +4—a theoretical, not necessarily observed in this review range, *Student‐Centered* is much greater than *Teacher‐Centered*).

In all, 365 effect sizes are included in the meta‐analysis. Four very large effect sizes (>4.00) are adjusted (Winsorized; Hastings, Mosteller, Tukey, & Winsor, 1947) to match the next lower effect size, the fifth largest, in the distribution (*g* = 3.1). This produces a change in the mean effect size of 0.10 and a similarly slight adjustment to the other statistics. There are no outliers at the negative end of the distribution.

The results of this analysis (See Table 6 for both the unadjusted and adjusted statistics) produces a significant weighted adjusted average effect size of $\bar{g}$ = 0.444, *k* = 365, *SE* = 0.03, *z* = 14.56, *p* < .000. The distribution is significantly heterogeneous (*Q*‐Total = 3095.89, *df* = 364, *p* < .0001, with an *I* ^2^ value of 88.22 and a *τ* ^2^ (tau‐squared) of 0.27. This result suggests that on average *more Student‐Centered* classroom studies produce better results on achievement outcomes than do *less Student‐Centered* classroom studies. The average weighted effect size is of moderate size (Cohen, 1988) and indicates that on average the more C‐S condition (treatment) outperformed the less C‐S (control) by 0.444*sd.* The average effect size is used as a reference point to describe the collection of student‐centered versus teacher‐centered practices when compared and reflects the overall benefit for learning when student‐centered qualities are present. The subsequent moderator variable analysis (presented in the next section) attempts to explain the extent to which each dimension contributes (or does not contribute) to the overall average.

**Table 6:** Overall results for unadjusted and Winsorized data sets

Model	Effect size and 95th confidence interval					Test of null	
Random effects	*k*	$\bar{g}$	*SE*	Lower 95th	Upper 95th	*z*‐value	*p* value
Unadjusted	365	0.454	0.03	0.39	0.52	14.44	<.00
Winsorized	365	0.444	0.03	0.38	0.50	14.56	<.00

Model	Heterogeneity				
Fixed effect	Q‐value	*df*	*p* value	*I* ^2^	*Tau* ^2^
Unadjusted	3,327.78	364	<.00	89.03	0.29
Winsorized	3,095.89	364	<.00	88.22	0.27

Simple meta‐regression can provide a sense of this relationship between the strength of *Student‐Centered* and achievement outcomes. A moderator variable reflecting the degree of *Student‐Centered* (the quantitative differences between the ratings of the treatment/control) was created to test this relationship. If this relationship is patterned (either positively or negatively) rather than irregular, the result of the meta‐regression of achievement on the degree of student centeredness should result in a positive, significant slope. If the slope of the regression line is not positive and significant, indicating the absence of a positive linear progression, we can assume that the relationship between student centeredness and achievement results is irregular, thereby diminishing the argument that more *Student‐Centered* classrooms are more advantageous to the attainment of achievement outcomes than less *Student‐Centered* Classrooms.

The simple meta‐regression of the relative difference between more *Student‐Centered* and less *Student‐Centered* resulted (the defining characteristic of the treatment‐control contrast) in a significant slope (*β* = 0.037, *SE* = 0.017, *z* = 2.14, *p* = .03). The test of the model resulted in *Q‐*Between = 4.58, *df* = 1, *z* = .03. *Q*‐within is also significant. These results indicate a marginally positive relationship between the degree of student centeredness and the achievement of learning outcomes by students. At best, this result is considered to be a weak but positive effect. Complete results of this analysis can be found in Table 7.

**Table 7:** Overall strength of the relationship between treatment and control (degree of student‐centeredness)

Covariate	*β*	*SE*	Lower 95th	Upper 95	*z*‐value	*p* value	VIF
Intercept	0.34	0.06	0.22	0.45	5.76	<.001	3.67
Degree of *Student‐Centered* instruction	0.04	0.02	0.00	0.07	2.41	.032	1.00
Test of model: Q = 4.58, *df* = 1, *p* = .032. Goodness of Fit: *Q* = 3094.41, *df* = 363, *p* < .000, *tau* ^2^ = 0.27							

*Note*: This and all subsequent meta‐regression analyses use Random Effects Method‐of‐Moments Model.

#### 5.3.2 Primary predictor variables

There are four primary predictor variables that represent the degree of the *Teacher's Role*, *Pacing*, *Flexibility*, and *Adaptivity* that is offered to students. Studies are coded as a differential between the treatment and control conditions in terms of less flexible/adaptive classroom practices or more flexible/adaptive practices. These differentials form a hypothetical continuous integer‐level scale ranging from −4 to −1 for *more Teacher‐Centered practices* (less flexible/adaptive) and +1 to +4 for *more Student‐Centered practices* (more flexible/adaptive), with 0 (zero) interpreted as equality between the control and treatment conditions. Please, keep in mind that though negative‐to‐positive fluctuations within each dimension are theoretically possible, only studies whose total differential score (the sum of four dimensions) is positive (i.e., overall in favor of *Student‐Centered* qualities of instruction) were retained in our meta‐analysis. There are four dimensions of classroom practice (these are described in detail in the Method) that were identified and to which this coding was applied:

- *Teacher's role* as a lecturer/guide/mentor;
- *Pacing* of instruction to meet student needs/preferences;
- *Flexibility* in the creation/use of study materials, course design, etc.;
- *Adaptability* of feedback and learning activities to students, individual interests of students, etc.

The question being asked in this moderator variable analysis is which, if any, of these classroom practices, predicts levels of effect size. Initially, meta‐regression is used to explore this question. Then, treating the scale as categorical data, the various levels are explored through mixed moderator variable analysis (i.e., *ANOVA‐*analog). Finally, combinations of these dimensions are explored to determine if they can better characterize the totality of the instruction.

##### Meta‐regression of dimensions (primary moderator variables)

Initially, all four dimensions were entered into multiple meta‐regression (random effects method of moments) in the order that they are described above. The dependent or outcome variable in this analysis was the effect sizes of individual studies (*k* = 365).

The overall model, excluding the intercept (See Table 8a), was significant (*Q*‐Between = 31.02, *df* = 4, *p* < .001). The extent of unexplained variation goodness of fit test for heterogeneity was also significant (*Q*‐within = 2,912.94, *df *= 360, *p* < .001, *I* ^2^ = 87.64%, *Tau* ^2^ = 0.2615, *Tau* = 0.5113). Two of the four moderator variables were significant predictors of effect size (though acting in opposite directions): *Pacing* (*β *= −0.1542, *SE* = 0.045, *z* = −3.45, *p* = .0006); and *Teacher's role* (*β* = 0.154, *SE* = 0.039, *z* = 3.96, *p* = .0001). The other two predictor variables, *Flexibility* and *Adaptability*, were not significant.

**Table 8:** a–b Meta‐regression results for interval‐level moderator variables

Covariates		*β*		*SE*		Lower 95th		Upper 95		*z*‐value		*p* value		VIF
**Meta‐regression of four predictors—a**														
Intercept		0.33		0.06		0.21		0.45		5.38		<.001		4.11
Pacing		−0.15		0.04		−0.24		−0.07		−3.45		<.001		1.04
Teacher's role		0.15		0.04		0.08		0.14		3.96		<.001		1.06
Adaptability		0.06		0.04		−0.02		0.14		1.52		.13		1.04
Flexibility		0.04		0.04		−0.04		0.11		0.99		.32		1.07
Test of model: Q = 31.02, *df* = 4, *p* < .001. Goodness of Fit: *Q* = 2921.91, *df* = 360, *p* < .0001, *tau* ^2^ = 0.51														
**Meta‐regression of two predictors (reduced model)—b**														
Intercept		0.36		0.06		0.25		0.47		6.21		<.001		3.74
Pacing		−0.14		0.04		−0.23		−0.05		−3.18		.002		1.01
Teacher's role		0.17		0.04		0.09		0.24		4.42		<.001		1.00
Test of model: Q = 27.74, *df* = 2, *p* < .001. Goodness of Fit: *Q* = 2920.95, *df* = 362, *p* < .0001, *tau* ^2^ = 0.51														

Abbreviation: VIF: variance inflation factor.

**Figure 4:**
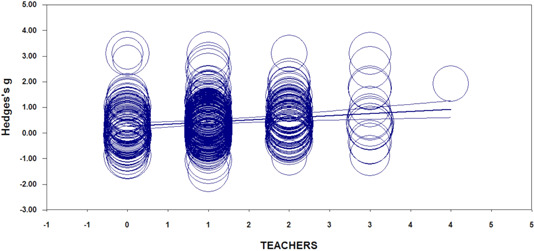
Scatterplot of Teacher's Role from meta‐regression (Table 7b). [Color figure can be viewed at wileyonlinelibrary.com]

**Figure 5:**
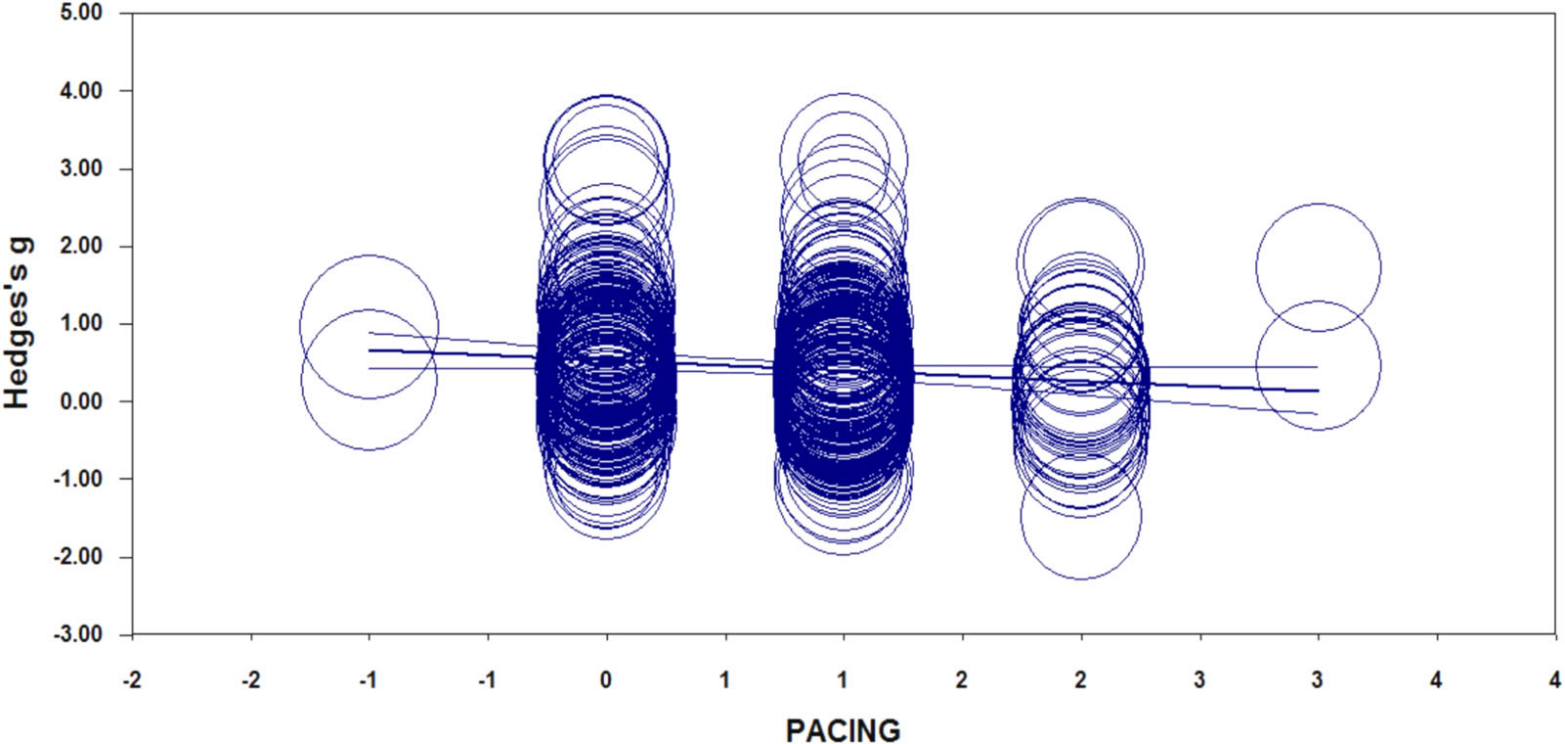
Scatterplot of Pacing from meta‐regression (Table 7b). [Color figure can be viewed at wileyonlinelibrary.com]

The analysis was re‐run (See Table 8b) with the two nonsignificant variables removed. This model was also significant (*Q* = 27.74, *df* = 2, *z* = 4.42 *p* < .001) and heterogeneous (*Q*‐within = 2,920.95, *df *= 362, *p* < .001, *I* ^2^ = 87.61%, *Tau* ^2^ = 0.2583, *Tau* = 0.508). The variables in the reduced model were both significant: *Pacing* (*β* = −0.139, *SE* = 0.044, *z* = −3.16, *p* = .0015); and *Teacher's role* (*β* = 0.167, *SE* = 0.038, *z* = 4.42, *p* < .0001). It is interesting that the moderator *Pacing* is a negative predictor of effect size, while *Teacher's role* is positive. Note that in all of these analyses the variance inflation factor is low, indicating a lack of collinearity (Thompson & Higgins, 2002). See Figures 4, 5 for scatterplots of these results.

To examine these results from another perspective, mixed moderator variable analysis was conducted for *Pacing*, *Teacher's Role, Adaptability, and Flexibility*, each explored across levels of differential scores (Table 9a–d). Several categories with cell frequencies (number of cases per level of the category) less than 5 were removed from each of these analyses. The same variables, *Pacing*, *Teacher's Role,* are significant across the categories of relative scores and the same pattern of —*Teacher's Role*, as a positive predictor of effect size, and *Pacing* as a negative predictor of effect size.

**Table 9:** a–d Comparison of levels of relative strength (how much Student‐Centered) for four primary moderator variables

Levels	*k*		$\bar{g}$	*SE*	Lower 95th	Upper 95th	*z*‐value	*p* value	*Q*‐Bet.	*df*	*p* value
**Teacher's Role—a**											
**0** (*Student‐Centered* =* Teacher‐Centered*)	80		0.31	0.07	0.17	0.44	4.51	.00			
**1** (*Student‐Centered* > *Teacher‐Centered*)	189		0.41	0.04	0.33	0.49	9.68	.00			
**2** (*Student‐Centered* >> *Teacher‐Centered*)	79		0.58	0.06	0.46	0.69	10.07	.00			
**3** (*Student‐Centered* >>> *Teacher‐Centered*)	15		0.78	0.21	0.37	1.20	3.72	.00			
Total between									12.65	3	.01
**Pacing—b**											
**0** (*Student‐Centered* = *Teacher‐Centered*)	151		0.54	0.05	0.44	0.64	10.59	.00			
**1** (*Student‐Centered* > *Teacher‐Centered*)	172		0.40	0.04	0.32	0.48	9.58	.00			
**2** (*Student‐Centered* >> *Teacher‐Centered*)	38		0.22	0.08	0.06	0.38	2.72	.01			
Total between									12.37	2	.002
**Adaptability—c**											
**0** (*Student‐Centered* = *Teacher‐Centered*)	179		0.38	0.04	0.31	0.46	9.94	.00			
**1** (*Student‐Centered* > *Teacher‐Centered*)	140		0.49	0.06	0.38	0.60	8.57	.00			
**2** (*Student‐Centered* >> *Teacher‐Centered*)	39		0.55	0.10	0.36	0.73	5.66	.00			
**3** (*Student‐Centered* >>>* Teacher‐Centered*)	6		0.41	0.15	0.12	0.70	2.77	.01			
Total between									3.99	3	.26
**Flexibility—d**											
**0** (*Student‐Centered *= *Teacher‐Centered*)	261		0.42	0.03	0.35	0.48	12.23	.00			
**1** (*Student‐Centered* > *Teacher‐Centered*)	64		0.47	0.09	0.29	0.65	5.18	.00			
**2** (*Student‐Centered* >> *Teacher‐Centered*)	29		0.60	0.11	0.40	0.81	5.76	.00			
**3** (*Student‐Centered* >>> *Teacher‐Centered*)	10		0.61	0.25	0.11	1.10	2.41	.02			
Total between									3.48	3	.32

There is nothing unique in this later analysis, but it does give a clear sense of the magnitude of effect within each category of relative strength. For example, *Teacher's Role* ranges in effect size magnitude from $\bar{g}$ = 0.78 at strength level 3 to $\bar{g}$ =  0.31 at level 0 (Table 9a), where the treatment and control conditions are equally balanced. *Pacing* (Table 9b) moved in the opposite with and average effect at strength 2 of $\bar{g}$ = 0.22 and at $\bar{g}$ = 0.54 at strength level 0. Both of these variables are significant in this analysis of categories.

By contrast, neither of the other two variables (Table 9c,d), *Adaptability* and *Flexibility*, is patterned or significant across levels of relative strength. For the two variables, average effect sizes ranges from $\bar{g}$ = 0.38 to $\bar{g}$ = 0.61, a relatively short range compared to *Teacher's Role* and *Pacing.*

The next question we asked concerns the combinations of these four variables. In this analysis, dimensions alone are compared with the dimensions paired (i.e., *Teacher's Role* + *Pacing*). The two significant predictors, *Teacher's Role* and *Pacing* are shown in Table 10. The between‐group *z‐*value comparing the two alone and their pair is not significant (*p* = .44).

**Table 10:** Teacher's role and pacing and their combination

Levels	*k*	$\bar{g}$	*SE*	Lower 95th	Upper 95th	*z*‐value	*p*‐value	*Q*‐Bet.	*df*	*p* value
**Teacher's role, pacing and combination**										
Teacher's role	61	0.48	0.07	0.34	0.62	6.83	.00			
Pacing	5	0.50	0.15	0.20	0.80	3.26	.00			
Teacher + Pacing	14	0.72	0.25	0.23	1.20	2.91	.00			
Total between								2.69	3	.44

However, when the other two variables, *Flexibility,* and *Adaptability*, are examined in the same way (single dimensions and pairs), the between‐group *z*‐value is significant (*p* = .01; See Table 11a). The two extremes with reasonable cell frequencies are further tested in *post hoc* analysis in Table 11b. These are *Teacher's Role* paired with *Flexibility* (*k* = 44, $\bar{g}$ = 0.31) and *Teacher's Role* paired with *Adaptability* (*k* = 33, $\bar{g}$ = 0.66). The overall *Q‐*between is significant (*z* = 7.58, *p* = .006).

**Table 11:** a–b Two variables (adaptability and flexibility) and their combinations

Levels	*k*	$\bar{g}$	*SE*	Lower 95th	Upper 95th	*z*‐value	*p* value	*Q*‐Bet.	*df*	*p* value
**Adaptability, Flexibility and combinations—a**										
Adaptability	19	0.23	0.11	0.02	0.44	2.10	.04			
Flexibility	29	0.19	0.09	0.02	0.35	2.17	.03			
Flexibility + Adaptability	15	0.35	0.24	−0.12	0.82	1.47	.14			
Flexibility + Teacher's role	44	0.31	0.08	0.15	0.46	3.89	.00			
Adaptability/Pacing	6	0.34	0.28	−0.21	0.89	1.20	.23			
Adapt. +Teacher's role	33	0.66	0.10	0.46	0.86	6.57	.00			
Total between								14.55	5	.01
**Teacher's role and combinations with Flexibility and Adaptability (post hoc)—b**										
Teacher's role	61	0.48	0.07	0.34	0.62	6.83	.00			
Flexibility + Teacher's role	44	0.31	0.08	0.15	0.46	3.89	.00			
Adapt. + Teacher's role	33	0.66	0.10	0.46	0.86	6.57	.00			
Total between								7.76	2	.02

*Note*: The total number of all single dimensions and combinations is *k* = 365. Some have been excluded (*k* = 226).

##### Demographic moderator variable analysis

Moderator variables in this study (Table 12a–d), beyond those already described, are mostly demographic in nature. Thus, they are less important to the main focus but they do give a sense of the range of conditions that exist within the data set. In Table 12a–d, it is interesting that only one demographic variable is significant—*Ability Profile* and that only one is close to significance (*STEM* vs. *Non‐STEM*; Table 12b). The first is a contrast of two categories with reasonable cell frequencies greater than five, *General Population* and *Special Education.* The effect sizes were $\bar{g}$ = 0.42 (*k* = 338) and $\bar{g}$ = 0.80 (*k* = 26) in favor of *Special Education.* Non‐STEM subjects outperformed STEM in absolute magnitude, but the contrast was not significant ($\bar{g}$ = 0.52 vs. 0.40).

**Table 12:** a–d Mixed moderator variable analysis for categorical demographics

Levels	*k*	$\bar{g}$	*SE*	Lower 95th	Upper 95th	*z*‐value	*p* value	*Q*‐Bet.	*df*	*p* value
**Grade Level—a**										
Kindergarten	7	0.44	0.18	0.10	0.79	2.52	.01			
Gr. 1–5	116	0.36	0.04	0.28	0.45	8.38	.00			
Gr. 6–8	95	0.47	0.07	0.34	0.60	6.93	.00			
Gr. 9–12	124	0.50	0.06	0.38	0.62	8.15	.00			
Total between								4.11	3	.39
**Subject matter (Non‐STEM vs. STEM)—b**										
Non‐STEM	93	0.52	0.07	0.39	0.65	7.93	.00			
STEM	260	0.40	0.03	0.33	0.46	11.36	.00			
Total between								2.80	1	.09
**Subject matter (Detailed)—c**										
ICT	13	0.36	0.17	0.03	0.69	2.17	.03			
Language Arts	52	0.48	0.06	0.35	0.60	7.57	.00			
Math	80	0.37	0.06	0.26	0.48	6.61	.00			
Science	168	0.43	0.05	0.33	0.52	8.94	.00			
Second Lang.	12	0.57	0.18	0.21	0.92	3.11	.00			
Social Sciences	12	0.64	0.27	0.03	0.69	2.33	.02			
Total between								2.97	5	.70
**Ability Profile—d**										
General Pop.	338	0.42	0.03	0.36	0.48	13.45	.00			
Special Ed.	26	0.80	0.15	0.50	1.10	5.24	.00			
Total between								5.96	1	0.01

To reiterate, complete descriptive statistics are contained in Table S13.

## 6 DISCUSSION

The purpose of this review is to examine the effectiveness of *Student‐Centered* instructional practices in k‐12 classes as it increases or depresses student achievement. Additionally, the study examines *four dimensions* of instructional practice, namely, “Teacher's role,” “Pacing of instruction,” “Flexibility of instructional activity,” and “Adaptability of instruction” for their individual and/or collective influence. In addition, five demographic moderator variables are also examined for their potential relationship to the effectiveness of *Student‐Centered* instruction.

### 6.1 Summary of main results

#### 6.1.1 Overall tests of *Student‐Centered* instruction

Two tests are used to judge the overall effectiveness of *Student‐Centered* instructional practices in promoting achievement outcomes in k‐12 learners.

- The first is a test of the overall outcome of 365 effect sizes. The results are significant, producing an average random effect of $\bar{g}$ = 0.444. Interpreted in terms of the normal distribution, this amounts to an increase for the more *Student‐Centered* condition of 17.5% (67.5 – 50 = 17.5) over the less *Student‐Centered* control condition. This result would be considered a moderate effect according to Cohen's (1988) interpretative criteria.
- The second is the result of simple meta‐regression of effect size on the total number of 0 to 4 codes for four dimensions for each study (e.g., ***P**acing*‐***F**lexibility*‐***T**eacher's role*‐***A**daptability* or 1‐2‐2‐1 = 6, the sum of differential scores across dimensions). Each study was represented by a number with a theoretical range of 0–16. The analysis resulted in a positive and significant relationship (*p* = .03) suggesting that as *Student‐Centered* totals increase, so does effect size. Taken together, these results reveal a tendency towards an advantage for more *Student‐Centered* practices compared with less *Student‐Centered* practices.

#### 6.1.2 Primary moderator variables

The next question relates to the four dimensions, represented by the codes for each dimension above disassembled from the total number referred. Each code ranges from 0–4 In the example above, this would give ***P**acing* a 1, ***F**lexibility* a 2, ***T**eacher's role* a 2, and ***A**daptability* a 1.

- The four dimensions are tested as predictors of effect size using multiple meta‐regression. Two dimensions are significant, *Teacher's role,* and *Pacing*; *Adaptability* and *Flexibility* were not.
- A second multiple meta‐regression, including only *Teacher's role* and *Pacing* also produces a significant overall result. However, the relationship between the two predictors is opposite: *Teacher's role* is significant and positive, whereas *Pacing* is significant and negative.
- The combination of these dimensions, tested using mixed moderator variable analysis reveals that *Teacher's role* and *Adaptability* is a better combination for promoting better *Student‐Centered* achievement than *Teacher's role* and *Flexibility* (i.e., compared in post hoc analysis). This combination also exceeds the overall average effect size for *more* Student‐Centered versus *less Student‐Centered* instruction ($\bar{g}$ = 0.66 vs. $\bar{g}$ = 0.44).

#### 6.1.3 Demographic moderator variables

Four demographic moderator variables were coded and the results of their analyses are described below.

- Three of the moderator variables are not significant: *Grade level*, *Subject matter* (i.e., *Non‐STEM* vs. *STEM courses*), and detailed Subject matter comparisons. None of these are significant across levels.
- The variable *Ability profile* is significant in between‐group analysis, with students in *Special Education* programs outperforming students deemed in the *General Population* (i.e., $\bar{g}$ = 0.80 vs. $\bar{g}$ = 0.42). This result seems not to be surprising, given that Special Education teachers are trained to provide individual attention to students in small classroom settings.

### 6.2 Overall completeness and quality of the evidence

Clearly, this database does not include every single classroom study since 2000 that tested two groups. To find, much less to process literature that is potentially as large as this would be is a monumental task. Therefore, we had to be selective and limit the database in two important ways. First, we selected only studies that contained two compared groups that included enough information in each group to assess the qualities of *Student‐Centered* that we were looking for. Second, we selected only high‐quality quasi‐experimental designs (QEDs) and randomized control trials (RCTs), thus further limiting the potential pool of studies. As a result, we consider this corpus of 299 studies and 365 independent effect sizes to be a reasonable representation of the larger body of studies that we either excluded or that could not be accessed.

### 6.3 Limitations and potential biases in the review process

#### 6.3.1 High‐inference versus low‐inference coding procedures

One of the obvious limitations and a potential source of bias in this study is the fact that it uses an extensive amount of *high‐inference* coding (Cooper, 2017). There is no treatment or control, per se, but instead, a set of judgments by reviewers, first as to the very definition of the treatment and control conditions (i.e., the treatment is the condition that is more *Student‐Centered* and, conversely, the control is the condition judged to be less *Student‐Centered*). These decisions by two independent coders were judged to be high in inter‐rater reliability for the direction of the effect (e.g., + vs. −; *κ* = >0.86), and for the precision of calculation (*κ *= >0.92). Second, judgments were made by coders as to the exact ratings (e.g., +3 vs. +4) applied to each of the four dimensions. Again, these decisions were made by at least two coders working independently and producing inter‐rater reliability of *κ *= 0.67. It is important to note in considering the accuracy of coding that raters/coders received extensive training for this task, including multiple practices runs on studies previously judged to have been accurately and reliably coded.

Also, it is worth noting that our research team has considerable experience with this approach to establishing the treatment and control through high inference coding, and have presented a paper on the subject at the Campbell Collaboration's Ninth Colloquium (Borokhovski, Bernard, Tamim, & Abrami, 2009) as well as included *high inference* coding in previously published meta‐analyses. In the earliest meta‐analysis (Bernard et al., 2009), we compared interaction treatments (i.e., practices that link students to each other, teachers. and content) in distance education to noninteraction treatments, and then classified them as student‐student, student‐teacher, student‐content interactions. The inter‐rater agreement for this exercise was *κ *= 0.71.

In a later meta‐analysis (Schmid et al., 2014) of the effects of technology treatments in postsecondary education, studies were rated for the degree of technology integration. Higher integration (i.e., longer, more extensive richer in functionality use of educational technology) was deemed the treatment and lower integration was the control. In this study, the inter‐rater reliability for this rating step was even higher (*κ *= 0.80), and in the same range as in the current study.

We recognize that this form of *high‐inference* coding contains greater risk of bias than the standard designation of treatment/control, which is normally referred to as *low‐inference* coding. However, we see no other way to advance research synthesis in literatures such as this one beyond relatively simple comparisons between “either this or that” comparisons like the treatment/control designations that populate the educational research literature. The alternative, of course, is for primary researchers to refine their questions, but that will take some time in coming.

### 6.4 Agreements and disagreements with other studies or reviews

This study is in strong agreement with much of the primary and secondary literature surrounding the question of the veracity of *Student‐Centered* educational practices (See in particular Table 1 for a summary of *Student‐Centered* related practices). Of the several meta‐analyses that have investigated the efficacy of active learning (i.e., operationalized here as more *Student‐Centered* learning) most have found a positive effect for it. In particular, reviews by Prince (2004), Linton, Farmer, and Peterson (2014), Burch et al. (2014), and Freeman et al. (2014) support the use of various *Student‐Centered* strategies in different levels of educational practice. However, ours is the only meta‐analysis that has approached the question in this fashion. It is also the only meta‐analysis that has examined where, in the range of instructional practices, this advantage for *Student‐Centered* instruction resides. Some of these reviews concern particular areas in postsecondary education (e.g., STEM subjects) and some are more general. The current review looks at STEM learning and individual studies beyond STEM. While not significantly different, these comparisons point to a generally positive effect across all subject areas covered in the corpus of the reviewed literature.

There are also reviews of direct instruction that have found that there are advantages for lecture‐based or *Teacher‐Centered* instruction (e.g., Stockard, et al. 2018) but it is arguable that there is a place for both forms of instruction and that it is an open question as to what the joint contributions of *Teacher‐Centered* and *Student‐Centered* are.

## 7 AUTHORS’ CONCLUSIONS

This meta‐analysis provides strong evidence that *Student‐Centered* instruction leads to improvements in learning with k‐12 students. Not only is the overall random effects average medium in magnitude ($\bar{g}$ = 0.44), but there is also a demonstrated (subtle but significant) linear relationship between more *Student‐Centered* classroom instruction and effect size (*p* = .03). Taken together, these results support the efficacy of allowing students to engage in active learning or other forms of *Student‐Centered* as part of a comprehensive educational experience. It does not, however, diminish the potential advantages imbued by direct instruction (i.e., *Teacher‐Centered* practices). Delivering important content and other kinds of directive information to students will always be part of ordered classroom processes. As Gersten et al. (2008) have argued, there is little evidence that classrooms are organized as purely *Teacher‐Centered* or *Student‐Centered*.

In regards to the principal moderator variables—*Teacher's role, Pacing, Flexibility,* and *Adaptability,* it is not surprising that *Teacher's role* occupies a central place in facilitating *Student‐Centered* classrooms and that the relationship of this variable to effect size produces a significant positive linear trend. It is less understandable why *Pacing* produces an effect in meta‐regression that is significantly negative. Apparently, the pacing of instructional events in a classroom is more productive when it is less *Student‐Centered* than when it is more *Student‐Centered*. It is possible that pacing is best left under the control of the teacher or at least mostly influenced by the teacher.

*Flexibility* and *Adaptability*, as tested in meta‐regression, failed to produce a linear relationship with average effect size. However, it is arguable that these variables are not primary, but may play a role in combination with *Teacher's Role* that either enhances or diminishes achievement outcomes. *Teacher's Role* plus *Adaptability* appears to boost average effect sizes, while *Teacher's Role* plus *Flexibility* appears to diminish the average effect size. This inverted relationship is not too hard to understand if one considers the definitions of the two dimensions as they were operationalized in this study:

- *Flexibility* is the individualized creation/use of study materials, course design; and
- *Adaptability* is the provision of feedback to students and learning activities that are geared to the individual interests of students.

*Flexibility* concerns the creation/choice of learning materials, a role that students do not often assume, and *Adaptability* is operationalized as consideration for individual students in terms of appropriately designed learning activities and individualized feedback on those activities. *Pacing*, found to be a negative predictor of achievement, does not appear to interact with *Teacher's Role.*

### 7.1 Implications for practice and policy

This study does not provide specific instructions for the design and development of more *Student‐Centered* Classrooms. However, besides the overall finding that more verses less *Student‐Centeredenteredness* improves achievement outcomes, it does suggest where these practices might be applied most beneficially in specific domains of practice. We understand from these results that the teacher's role in creating an *Student‐Centered* classroom is critical. Given more freedom to develop intellectually as an individual and with peers (i.e., any of a variety of group‐based approaches) does appear to lead to better achievement outcomes compared to more direct forms of *Teacher‐Centered* instruction. This is one lesson that is worth learning and enacting across the k‐12 spectrum since there was no differentiation among grade levels. Similarly, there was no distinction between STEM and Non‐STEM courses, nor were any of the individual subject matters reliably different from one another. This suggests a universal phenomenon that is even more pronounced in Special Education courses compared to the general population of students.

### 7.2 Implications for research

As we have in the past (Bernard et al., 2009; Schmid et al., 2014) we argue that new research efforts in classroom‐based research move toward to more nuanced questions concerning practice. So much of classroom instruction asks the question, “Does alternative treatment X outperform classroom instruction or traditional educational practices.” There were a time and place for this either/or form of research, but as the efficacy of instructional approaches is validated, new questions about varieties of the new treatments need to be asked and answered. This quote from Bernard, Borokhovski, and Tamim (2019) expresses this sentiment in clear terms: “To use David Cook's (2009) analogy, can you imagine how far automobiles would have developed if they had always been compared to their reasonable alternative at the turn of the 20th century, the horse?” In spite of Henry Ford's declaration that “if I'd listened to my consumers, I'd have given them a faster horse” (Sherrington, 2003, p. 8), the driving public got something much better than a faster horse, largely because it was abandoned as the comparison condition (p. 20). Educational researchers should do the same.

## INFORMATION ABOUT THIS REVIEW

### Roles and responsibilities

The review team on this meta‐analysis possesses the breadth and depth of experience suggested by the Campbell Collaboration. The recommended optimal review team composition includes (a) *at least one person on the review team who has content expertise* (Bernard has 7 years of elementary education experience, three in a *Student‐Centered* school; Schmid is an Educational Psychologist focusing on instructional methods, and Waddington is an Educational Philosopher focusing on constructivist and *Student‐Centered* theory), (b) *at least one person who has methodological expertise* (Bernard and Borokhovski have authored and co‐authored many published meta‐analyses, including five published in *Review of Educational Research*), and conduct workshops in M‐A. methodology), *and* (c) *at least one person who has statistical expertise* (Bernard and Borokhovski both have extensive statistical experience and Bernard has taught statistical methods to M.A. and Ph.D. students for over 20 years). *It is also* (d) *recommended having one person with information retrieval expertise* (Pickup possesses an MLIS degree from McGill University, has been involved in retrieval and data management for our systematic review team for 5 years, and works as a methods reviewer for the Campbell Collaboration).

**Responsibilities:**

● **Content:** Robert M. Bernard, Eugene Borokhovski, Richard F. Schmid, and David I. Waddington
● **Systematic review methods:** Eugene Borokhovski and Robert M. Bernard
● **Statistical analysis:** Robert M. Bernard and Eugene Borokhovski
● **Information retrieval:** David Pickup
● **Update and revision:** David Pickup and Eugene Borokhovski

### SOURCES OF SUPPORT

Bernard, R. M. [PI], Borokhovski, E., Schmid, R. M., Waddington, D. I., & Pickup, D. (2016–2018). A Meta‐Analysis of 21st Century Adaptive Teaching and Individualized Learning Operationalized as Specific Blends of Student‐Centered Instructional Events. Jacobs Foundation and the Campbell Collaboration. Support: $50,000USD.

### PLANS FOR UPDATING THE REVIEW

This review will be updated on an annual basis.

### DECLARATIONS OF INTEREST

None of the authors are in conflict of interest with the goals or the outcomes of this meta‐analysis.

Publication bias analysis (See Figures 3–5 and Tables 1–4)

Methodological comparisons (sensitivity analysis; See Tables 5a–e)

Overall results (See Tables 6 and 7)

Results: Substantive moderator variables (See Tables 8a–b, 9a–d, 10, and 11a–b)

Demographic moderator variables (See Tables 12a–d)

## Supporting information

Supplementary information
