# Supplementary material for: Twenty‐first century adaptive teaching and individualized learning operationalized as specific blends of student‐centered instructional events: A systematic review and meta‐analysis
Source: Campbell Syst Rev. 2019 Jul 19;15(1-2):e1017. doi: 10.1002/cl2.1017 (PMC8356521; doi:10.1002/cl2.1017)
Supplement: Supplementary file 1 — Supplementary information [file CL2-15-e1017-s001.docx]

# Online supplements

List of online supplements

Online supplement 1 – References to Excluded Studies

Online supplement 2 – Table 13 (Descriptive statistics for each study)
